# Acquisition of new function through gene duplication in the metallocarboxypeptidase family

**DOI:** 10.1038/s41598-023-29800-9

**Published:** 2023-02-13

**Authors:** Daniel Fajardo, Ritchie Saint Jean, Peter J. Lyons

**Affiliations:** Department of Biology, Andrews University, Berrien Springs, MI 49104 USA

**Keywords:** Biochemistry, Evolution

## Abstract

Gene duplication is a key first step in the process of expanding the functionality of a multigene family. In order to better understand the process of gene duplication and its role in the formation of new enzymes, we investigated recent duplication events in the M14 family of proteolytic enzymes. Within vertebrates, four of 23 M14 genes were frequently found in duplicate form. While AEBP1, CPXM1, and CPZ genes were duplicated once through a large-scale, likely whole-genome duplication event, the CPO gene underwent many duplication events within fish and Xenopus lineages. Bioinformatic analyses of enzyme specificity and conservation suggested a greater amount of neofunctionalization and purifying selection in CPO paralogs compared with other CPA/B enzymes. To examine the functional consequences of evolutionary changes on CPO paralogs, the four CPO paralogs from *Xenopus tropicalis* were expressed in Sf9 and HEK293T cells. Immunocytochemistry showed subcellular distribution of Xenopus CPO paralogs to be similar to that of human CPO. Upon activation with trypsin, the enzymes demonstrated differential activity against three substrates, suggesting an acquisition of new function following duplication and subsequent mutagenesis. Characteristics such as gene size and enzyme activation mechanisms are possible contributors to the evolutionary capacity of the CPO gene.

## Introduction

Since Ohno’s seminal book^1^, gene duplication has been considered a major driving force in evolution, providing new material on which natural selection can act. Many studies have since provided more detail on how gene duplicates might arise, how they might be maintained and fixed in a population, and how they might develop new functions, distinct or complementary to the function of the progenitor gene^2–4^. For example, transposons are abundant mobile genetic elements that often copy DNA segments through RNA intermediates^5^. One variety of such retrotransposon, the LINE-1 retrotransposon, is active in the human genome today and is known to be responsible for disease-causing mutations^6^. DNA transposons that do not use an RNA intermediate are also abundant; a mechanism whereby some such elements complete this copying through a circular DNA intermediate has recently been proposed^7^. Recombination events that occur during both meiosis and mitosis can result in unequal crossing over and the tandem array of DNA segments^8^, while large-scale rearrangements such as chromosomal translocations result from DNA double-stranded break repair and non-homologous end-joining and are frequent occurrences that can lead to duplication of large segments of a genome^9^. Finally, whole genome duplication has occurred occasionally in metazoan lineages, particularly in plants^10,11^.

While it is clear that many genetic changes such as these occur throughout the natural history of a species, the selective forces that enable the maintenance of such changes are not always evident. Increased gene dosage is most often detrimental^12^. In some cases, random mutations occur following gene duplication, leading to a new function in one of the copies that benefits the organism (neofunctionalization). One example that has been described is the duplication of a sialic acid synthase gene in an Antarctic zoarcoid fish, leading to the evolution of an antifreeze protein^13^. In other cases, the function of a progenitor gene may be split between the resulting duplicate genes in terms of biochemical function or spatiotemporal expression, in a process that has come to be known as subfunctionalization. An example of this is the duplication of the human embryonic hemoglobin-γ gene, resulting in an additional form of hemoglobin expressed solely in the fetus^14^.

Many questions remain regarding how this process might work. Since it is rare that a double complement of gene expression is advantageous, and neutral evolution (gene drift) is most likely to result in rapid decay back to the original condition of one gene, what enables a duplicate gene to be maintained long enough for these neofunctionalization or subfunctionalization events to occur? Are particular biochemical features required in the gene to allow for increased gene dosage? Is a certain level of promiscuity required in the parental gene, or perhaps narrower specificity in the daughter genes?

**Figure 1:**
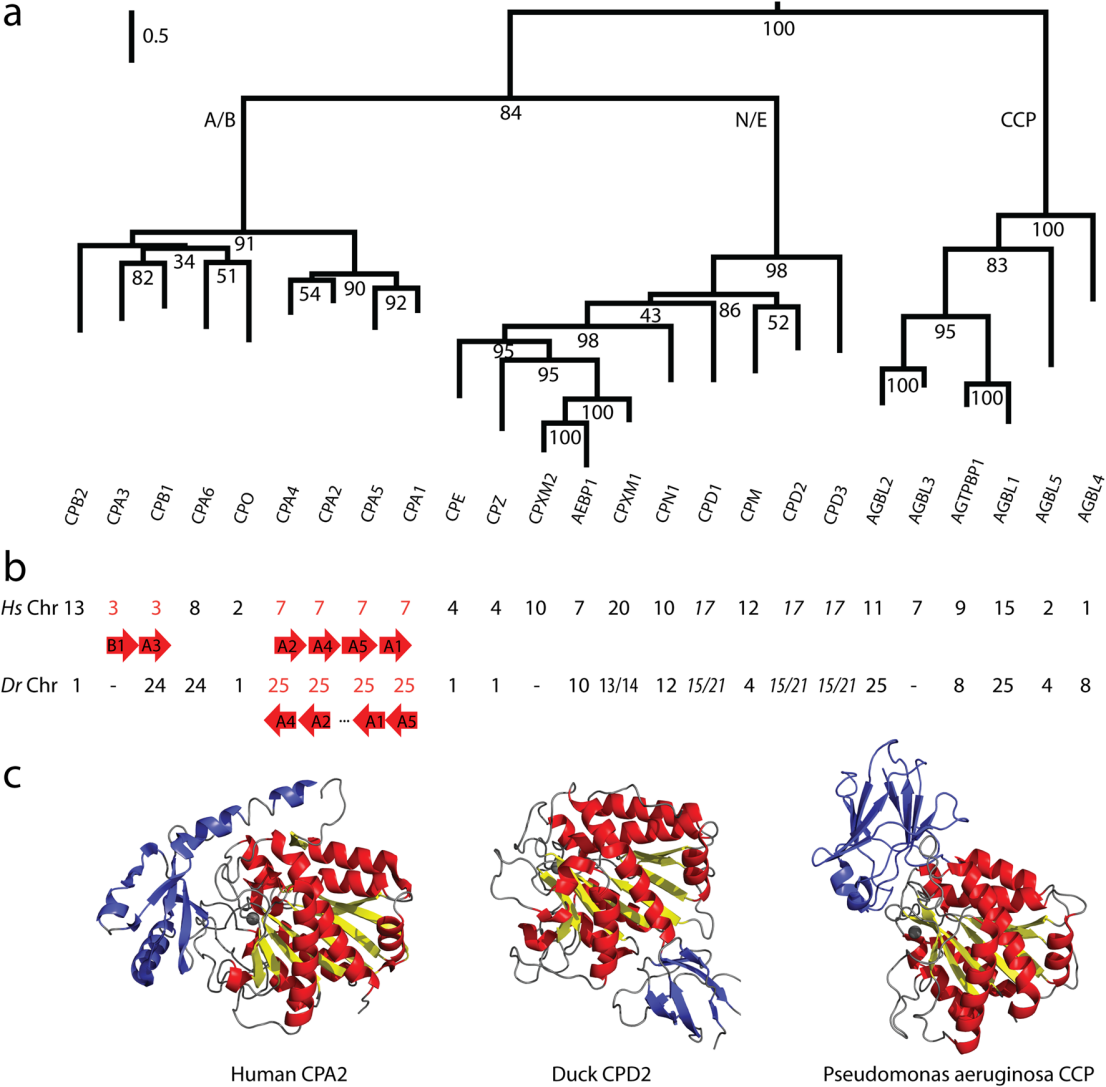
A history of gene duplication is shown by protein sequence similarities, gene synteny, and structural conservation within the M14 family of metallocarboxypeptidases. (**a**) A phylogenetic tree showing the relationships of the core catalytic domains of all human M14 proteins, clearly indicating the three major subfamilies (A/B, N/E, and CCP). Outgroups used for this tree were the aminoacylases, ASPA and ACY3, considered by some to be a fourth, although quite distant, subfamily of MCPs. Bootstrap values are shown at key nodes. Scale bar indicates substitutions per site. The CPD protein contains three tandem carboxypeptidase domains, hence this protein is represented by three branches. (**b**) The chromosomal location of each metallocarboxypeptidase gene. Those showing a tandem arrangement, suggestive of recent duplications, are shown in red with arrows below indicating their precise synteny. CPXM1 and CPD are duplicated in the zebrafish genome, thus two chromosomes are indicated. (**c**) Representative structures are shown for each of the three subfamilies of metallocarboxypeptidases, illustrating their homologous CP domains, yet different N- and C-terminal domains. Human CPA2 (1AYE) from the CPA/B subfamily, duck CPD2 (1QMU) from the CPN/E subfamily, and *Pseudomonas aeruginosa* cytosolic carboxypeptidase (4A37) representing the CCP subfamily.

The metallocarboxypeptidase (MCP) family of proteolytic enzymes is encoded by a large multigene family that arose through many of the duplication events described above. Each protein of this family contains, minimally, a carboxypeptidase enzymatic domain that is usually responsible for cleaving C-terminal amino acids, with many members also including one or two additional regulatory domains (Fig. 1A,C). The MCP protein family is most commonly described to have three subfamilies, named for the members that were first identified. The A/B subfamily contains the pancreatic enzymes CPA and CPB, which have played key roles in our understanding of hydrolytic mechanisms since the 1930s^15^, a great advantage when predicting enzyme function from sequence data. These enzymes typically contain an N-terminal inhibitory prodomain necessary for regulation of enzyme activity until needed^16,17^. Enzymes of the N/E subfamily are not regulated by a prodomain, but rather by pH and subcellular location. They commonly function within the secretory pathway in the maturation of propeptides such as neuropeptides or peptide hormones, or are secreted^18–20^. The third major subfamily of MCPs is the cytosolic carboxypeptidase (CCP) subfamily. These are expressed in the cytosol, where they have recently been found to process tubulins and other proteins^21–23^.

Evidence for specific duplication mechanisms can be observed occasionally within this family. For example, several genes encoding members of the A/B subfamily are found tandemly arrayed (Fig. 1B), suggestive of a meiotic crossing over error leading to their formation. Their continued close arrangement suggests a more recent duplication event than those leading to other genes of the family. Members of the A/B subfamily present different substrate specificities while maintaining very similar enzyme scaffolds (Fig. 1C). It has been shown that a simple change of residue 255 (numbering derived from bovine CPA1) at the base of the active site pocket results in a change in substrate specificity^24^, thus an apparently simple approach to neofunctionalization. The six CPA enzymes found in most vertebrates typically contain a Leu255 or Val255, resulting in the ability to cleave aliphatic or aromatic C-terminal amino acids. The two CPB enzymes contain an Asp255, resulting in the ability to cleave basic C-terminal amino acids. CPO contains an Arg255, thus providing acidic C-terminal substrate specificity.

In this paper we take advantage of abundantly available genomic data and a great depth of knowledge of MCP catalytic mechanism to consider recent duplication events within this MCP family of genes. Predicted protein sequences are analyzed and answers to some of the above-mentioned questions are suggested, at least within the context of this family. We find that four members of this family are further duplicated in many species, although not broadly across all phyla, suggesting more recent duplication events. While several of these gene duplications were likely a result of whole genome duplications or large-scale duplication events, CPO is frequently found duplicated within tandemly arrayed gene clusters. Our analysis suggests that many CPO paralogs are subjected to continuing purifying selection and that those present in *Xenopus tropicalis* have diversified in terms of function.

## Results

### Recent gene duplication in the MCP gene family

The MCP family of enzymes presents evidence of common ancestry, both in terms of sequence and structural homology and the chromosomal arrangement of genes. We were interested to know if any of these MCP genes continued to be duplicated more recently, and therefore if we could learn about mechanisms of duplication and subsequent selection through this larger dataset. Therefore, we explored the Ensembl database (release 98), containing genome sequence information for 188 vertebrate species, to identify vertebrate organisms containing multiple copies of these genes. This data was submitted to extensive manual analysis to ensure, as much as possible, that our conclusions were not based on incomplete and fragmented genomic data or incorrect genomic annotations.

**Figure 2:**
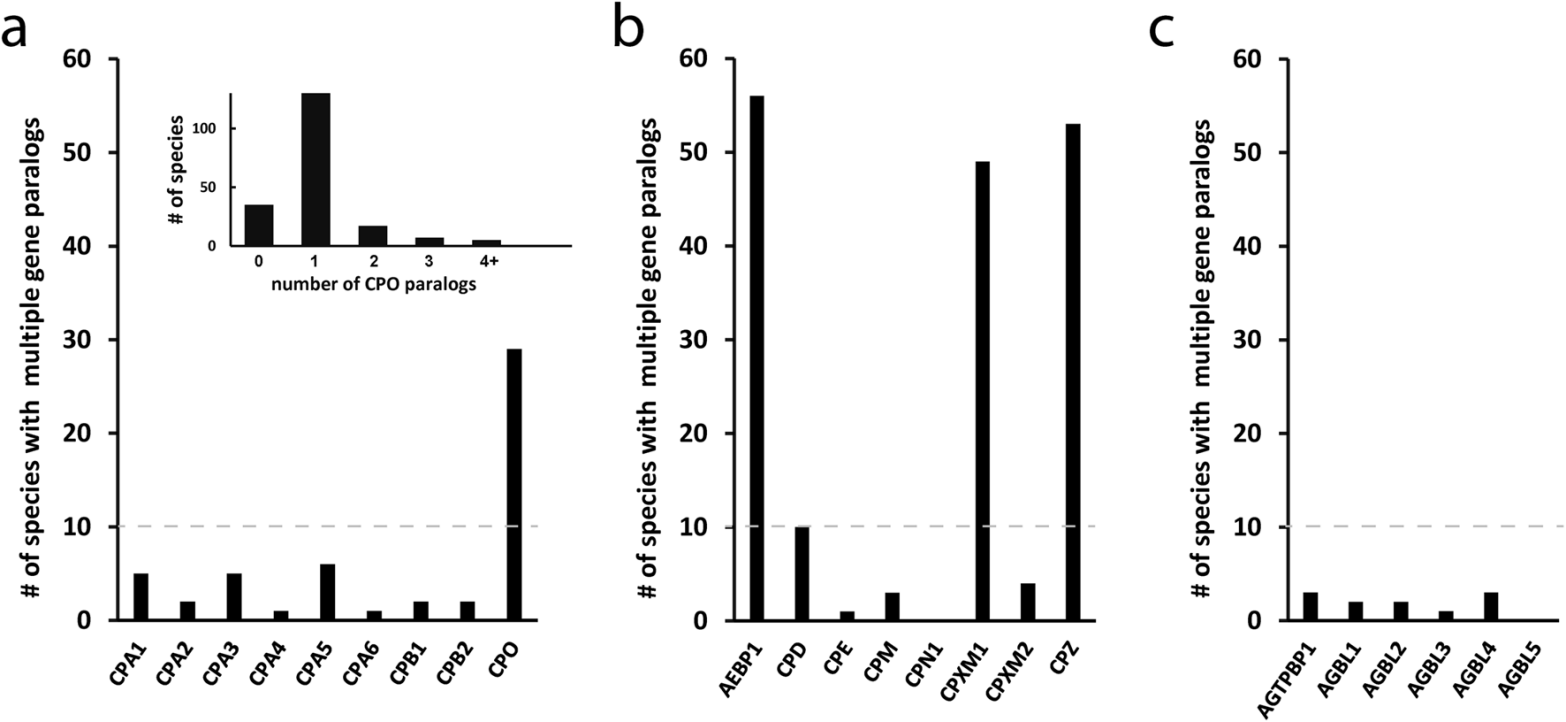
Several genes within the M14 family are further duplicated within many species. All orthologs of human M14 metallocarboxypeptidases of the (**a**) A/B subfamily, (**b**) N/E subfamily, and (**c**) CCP subfamily were identified in Ensembl Release 98. These were manually validated using information on gene synteny and completeness and updated with information from Ensembl Release 100.

While most MCP genes were present in duplicate form in a small number of species (1–10), several genes were found more frequently duplicated, such as the CPO gene within the A/B subfamily (Fig. 2). Notably, while a large majority of species contained only one CPO gene, and 29 vertebrate species contained two CPO genes, a number of species, including many, although not all, rodents, contained no functional CPO gene, but rather a pseudogene^25^. At the other extreme, some species, notably many fishes and *Xenopus tropicalis*, contained more than two copies of the CPO gene (Fig. 2A). A phylogenetic analysis of the proteins predicted to be translated by these CPO genes suggested a number of duplication events throughout the natural history of these species (see Supplemental Figs. S1 and S2 online). Evidence for gene conversion was found in a number of cases. For example, the zig-zag eel and swamp eel both contained 3 homologous CPO genes with very similar gene synteny, suggesting duplication events prior to the divergence of these two species; however, intraspecific homology between these protein sequences was greater than interspecific homology.

Within the N/E subfamily, AEBP1, CPXM1, and CPZ were found frequently in duplicate form (Fig. 2B). Nearly all ray-finned fishes (54 of 60 species) contained two AEBP1 paralogs, one annotated as aebp1 and the other as si:ch1073-459j12.1. The huchen appears to have an additional duplication of each of these genes. Only one other species in Ensembl98, the chimpanzee, exhibited a duplication of AEBP1 (upon manual analysis, others reported in R98 as duplicated were in fact two halves of the same gene). CPXM1 and CPZ present a similar story, in which 49 and 53, respectively, of 60 ray-finned fishes contained two paralogs (cpxm1a and cpxm1b, CPZ and cpz), with no duplicates present in other phyla (Fig. 2B). No CCP genes were found frequently in duplicate form (Fig. 2C).

### Gene synteny suggests duplication mechanisms

**Figure 3:**
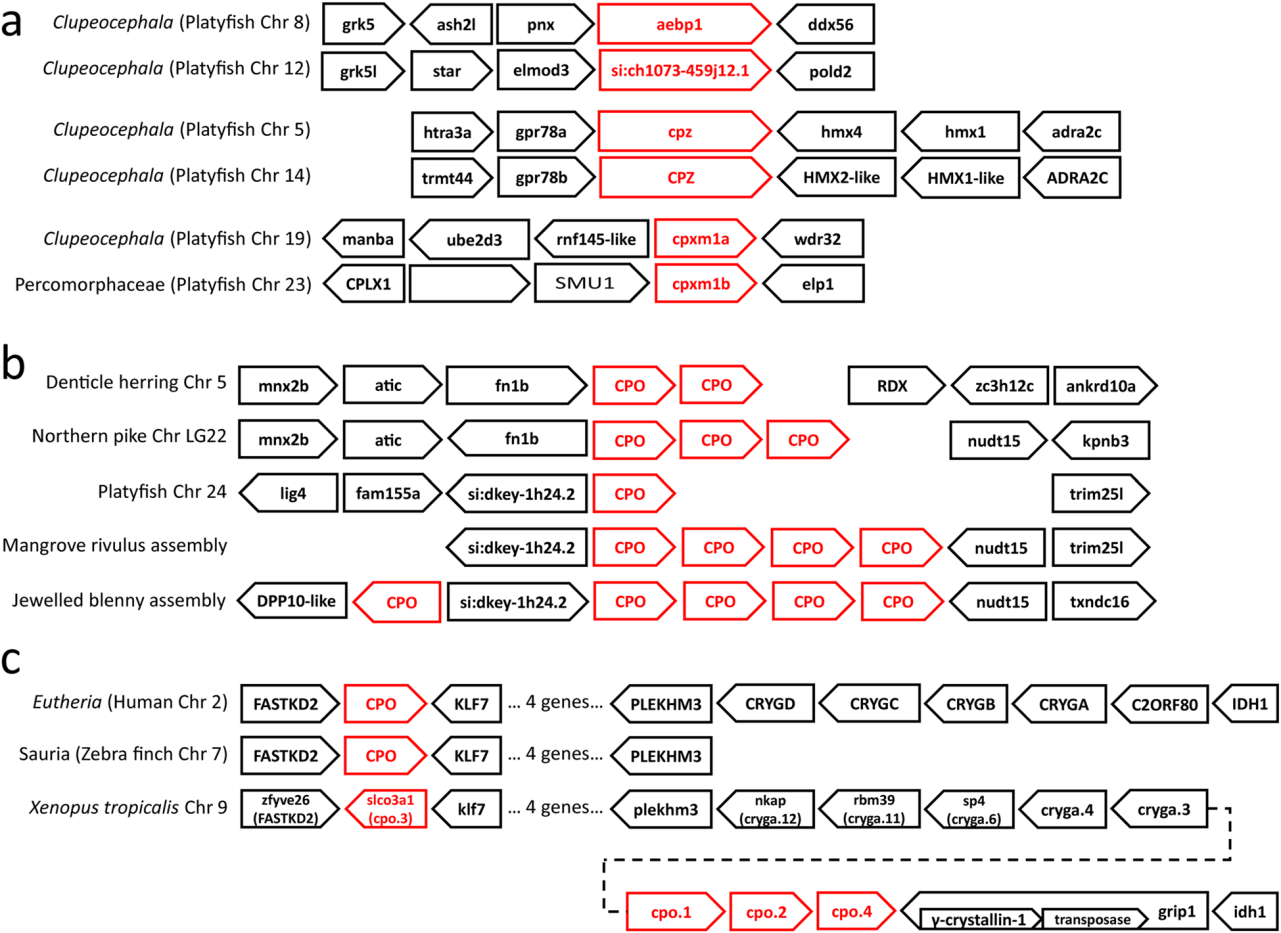
Gene synteny suggests that tandem CPO duplicates were formed through unequal crossing over. Gene synteny information was collected from Ensembl. (**a**) Aebp1, CPZ, and cpxm1 gene paralogs were found on different chromosomes within large blocks of common genes, suggesting a largescale duplication event. (**b**) CPO gene paralogs were always found in tandem, suggesting unequal crossing-over errors. (**c**) CPO gene paralogs in Xenopus tropicalis were found within a gamma crystallin gene cluster and just upstream of a transposase-like gene.

The presence of AEBP1, CPXM1 and CPZ gene duplicates throughout the ray-finned fish lineage suggested they were the result of a gene duplication event prior to the divergence of this group. The members of these gene duplicate pairs were on separate chromosomes and, in the cases of AEBP1 and CPZ, were surrounded by similar genes (grk5 and grk5l upstream of aebp1 duplicates, and gpr78-and hmx-like genes surrounding both cpz duplicates; Fig. 3A), suggesting that these duplication events impacted large chromosomal segments, possibly the result of a whole-genome duplication event^10^. Phylogenetic analysis confirmed this proposed relationship (see Supplemental Fig. S3 online).

In contrast, many CPO gene copies were arranged tandemly (Fig. 3B). Examples could be found of fish species with two to four tandemly arranged CPO genes, all with some level of shared syntenic relationships with neighboring genes, depending on evolutionary relationships of species. For example, many of these gene clusters were flanked by fn1b on one side and/or nudt15 on the other side (Fig. 3B). The Ensembl release 100 genome assembly for the jewelled blenny contained five CPO genes in close chromosomal proximity. The reason for this chromosomal amplification of the CPO gene within fish lineages was not apparent from a survey of the DNA sequences. Repetitive sequences were found in abundance throughout introns and intergenic sequence, although it was not clear that this was abnormal for such regions. However, the tandem arrangement of CPO genes was suggestive of an origin in unequal crossing over^8^.

An analysis of CPO gene synteny in the *Xenopus* lineage suggested a possible cause for duplication in this case. The parental CPO gene, that is, the CPO paralog found with identical synteny throughout non-fish vertebrates (eutherians, saurians, and amphibians), was found just upstream of the gamma-crystallin gene cluster in both eutherians and *Xenopus* (Fig. 3C). In *Xenopus tropicalis*, three additional copies of the CPO gene could be found between these gamma crystallin genes and the following grip1 gene. Within the last intron of this grip1 gene there were two additional annotated open reading frames, one predicted to encode a gamma-crystallin-1-like protein, and another with homology to DNA transposases (Fig. 3C). While DNA transposases are not commonly thought to be involved in this kind of tandem duplication, the observation of a CPO gene cluster together with a gamma-crystallin gene cluster and a putative transposase was suggestive of a link between all three. Examples of transposable elements involved in tandem duplication have been shown in maize^26^ and humans^27^.

### Retention of duplicated genes could be aided by reduced gene size

**Figure 4:**
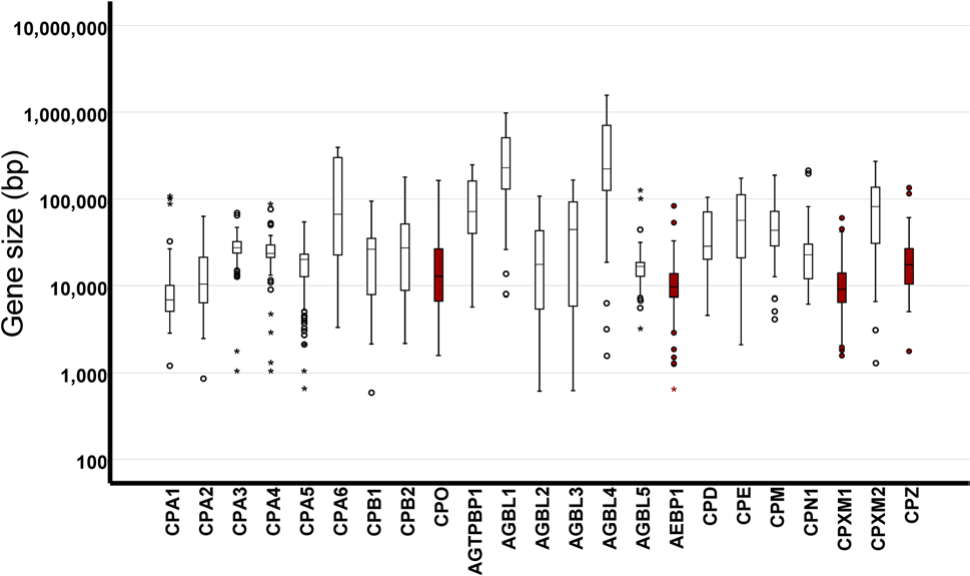
Gene size may contribute to rate of gene duplication or duplicate maintenance. The sizes of the indicated genes from 188 species were obtained from Ensembl (Release 98). An independent sample *t* test was performed comparing all frequently duplicated genes (red) to all others (white; *p* = 1.59E−37).

We wished to further investigate the reason for the unique expansion of, and particularly the retention of, CPO genes within fish and amphibian lineages. Gene synteny suggested an origin in meiotic crossing over events, with a possible role of a transposase in *Xenopus*. However, further selective pressures must be present to ensure maintenance of these genes in the genome. We considered that both the duplication of a gene and its retention in the genome could be influenced by gene size. Complete gene duplication could be aided by a smaller gene size, while smaller genes might avoid detrimental mutation longer than larger genes, resulting in their maintenance in the genome longer than larger genes. Therefore, MCP gene size was analyzed using information provided by Ensembl release 100. While gene size varied dramatically across phyla, a statistically significant reduction in gene size (*p* = 1.593 × 10^–37^) was observed for the four commonly duplicated MCP genes (CPO, AEBP1, CPXM1, and CPZ) when compared to the other MCP genes. The mean gene size for 1181 vertebrate CPO, AEBP1, CPXM1, and CPZ genes was 15,214 base-pairs, while that for the remaining 4120 vertebrate MCP genes found in the Ensembl database was 83,159 base-pairs (Fig. 4). While the number of genes analyzed here was minimal, this did suggest a role for gene size in this process. It is interesting to note that, while the CPXM1 and CPXM2 genes produce proteins with very similar structures and functions^28–30^, the CPXM1 gene is much smaller than CPXM2 and is maintained in duplicate form in fish genomes (Fig. 4; see also Figs. 2B, 3A).

Some studies have suggested that gene expression can affect the rate of gene evolution through dosage sensitivity^12^. Some genes are particularly sensitive to an increased expression level and so a second copy is rapidly purged from the genome. A recent study examined questions surrounding the impact of gene duplication on expression using a large comparative RNA-seq dataset^31^. However, there is limited data available on the impact of expression levels on gene duplication^32^. An analysis of human MCP gene expression did not reveal any unique characteristics in terms of gene expression for the CPO, AEBP1, CPXM1 and CPZ genes (see Supplemental Fig. S4 online).

### Duplicated MCP genes present evidence of neofunctionalization

Following gene duplication, selection has the opportunity to relax and explore new function through neofunctionalization or subfunctionalization. A wealth of information is available regarding MCP protein structure and enzymatic mechanism and this can allow us to make predictions regarding putative MCP gene function, whether it be the same function as the parental gene, neofunctionalized to produce an enzyme with a new substrate specificity, neofunctionalized to produce a protein with no enzyme activity (yet perhaps retaining another role in protein–protein interactions as a pseudoenzyme), or degraded to become a nonfunctional pseudogene. We focused on the CPA/B subfamily, as the tandem amplification and retention of CPO genes was of most interest.

We first set out to predict whether CPA/B genes found in duplicate form produced active enzymes, pseudoenzymes, or were unlikely to produce a functional protein at all (i.e. were pseudogenes). In order to classify predicted proteins in this manner, we needed to have an inventory of amino acid residues critical for carboxypeptidase structure or enzymatic function. To develop this inventory, we prepared a multiple alignment containing protein representatives of each member of the CPA/B subfamily from a broad range of taxa, typically including five mammal sequences (including one afrotherian, one marsupial, and one monotreme sequence), four sauropsid sequences (two bird and two reptile), one amphibian sequence, and two fish sequences. The resulting alignment indicated conservation of key catalytic residues that would be required for an active enzyme (H69, E72, and H196, involved in coordination of the required zinc ion; R127, N144, and R145, required for binding the carboxyl group at the C-termini of substrates; and E270, the key residue involved in acid–base catalysis^33^; see Supplemental Fig. S5 online), as well as many other residues conserved entirely, that are likely necessary for the structural integrity of these proteins (all amino acid numbers are derived from bovine CPA, as is the convention in this field). With this as a guide, we classified each predicted protein arising from duplicated genes as an active protein (all residues conserved in our pan-alignment are present), a pseudoenzyme (one or more residues necessary for enzyme activity are substituted, but other conserved residues retained), or a pseudogene (multiple conserved residues are substituted; see Supplementary Table S1 online). In some cases, predicted proteins were partial or fragmented in some manner that excluded key segments. Genes encoding these proteins were classified as pseudogenes, although in some cases they might simply be a result of incomplete genome sequencing or annotation.

**Figure 5:**
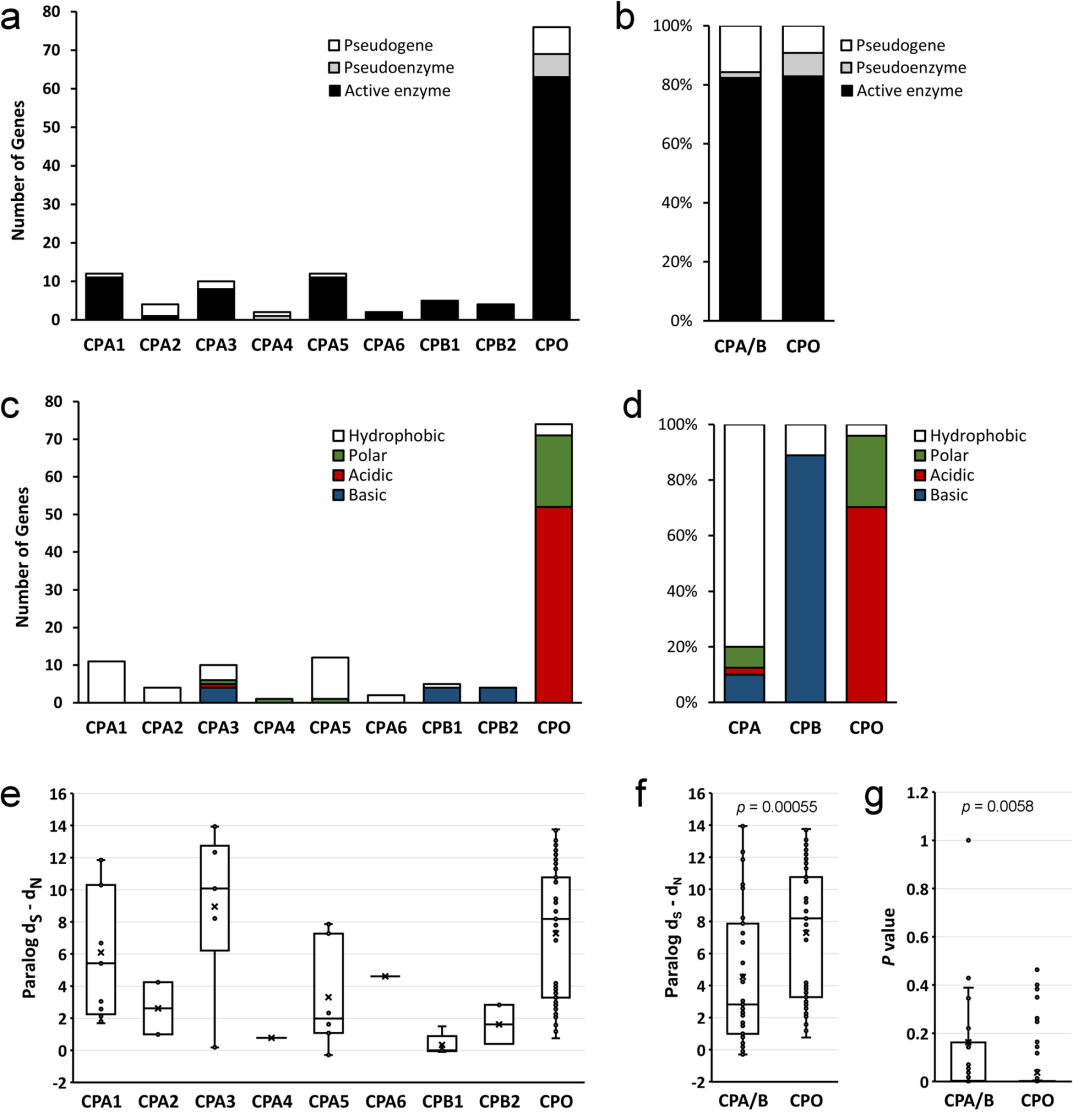
Indicators of gene function suggest changes in metallocarboxypeptidase paralog activity and specificity, yet purifying selection to maintain function. Predicted cDNA and amino acid sequences were curated for all duplicated members of the A/B subfamily of metallocarboxypeptidases found in Ensembl. (**a**, **b**) Predicted proteins were classified as active enzymes, pseudoenzymes (containing substitutions at active site residues), or pseudogenes (containing large deletions or other deleterious structural mutations). (**c**, **d**) Substrate specificity for each predicted protein was predicted based on the identity of the bovine CPA1 residue 255 equivalent. Hydrophobic = hydrophobic residue 255; acidic = basic residue 255; basic = acidic residue 255; polar = polar residue 255. (**e**, **f**) The predicted coding sequences for each paralog pair were used in a codon-based test of purifying selection, where greater Ds–dN indicates greater probability of purifying selection. The variance of the difference of synonymous and nonsynonymous substitutions per site was computed using the analytical method. Analyses were conducted using the Nei–Gojobori method in MEGA7. (**g**) The probability of rejecting the null hypothesis of strict-neutrality (dN = dS) in favor of the alternative hypothesis (dN < dS, purifying selection) is shown. A t-test was used to compare CPO paralogs with all others.

Following this approach, 83% (63/76) of all duplicated CPO genes, and 82% (42/51) of all other duplicated CPA/B genes (all CPA and CPB genes combined, as numbers of each were low), were predicted to encode active enzymes (Fig. 5A,B). Eight percent (6/76) of duplicated CPO genes were predicted to encode pseudoenzymes due to one or more substitutions in strictly conserved active site residues, in contrast to other CPA/B genes, in which only one pseudoenzyme was predicted (1/51). Likewise, most duplicated genes predicted to encode enzymes retained their expected substrate specificity: 80% (32/40) of CPA genes were predicted to encode enzymes with specificity to cleave aliphatic/aromatic C-terminal amino acids (having a hydrophobic residue at position 255) and eight of nine CPB genes were predicted to encode enzymes with specificity to cleave basic C-terminal amino acids (having an acidic amino acid at position 255; Fig. 5C,D). Only 70% (52/74) of CPO genes were predicted to encode enzymes with specificity to cleave acidic C-terminal amino acids (having a basic amino acid at position 255). However, many of the remaining CPO-like enzymes contained polar amino acids at residue 255 (26%; 19/74), with unknown impact on the substrate specificity of these enzymes.

In order to investigate the selective pressures placed on these genes, we compared the identified gene paralogs using the method of Nei and Gojobori to estimate synonymous (dS) and nonsynonymous (dN) nucleotide substitutions per site. The probability of rejecting the null hypothesis of strict-neutrality (dN = dS) in favor of the alternative hypothesis of purifying selection (dN < dS) was determined for each paralog pair. The resulting statistic (dS–dN) suggested widely varying levels of purifying selection for each gene within the A/B subfamily (Fig. 5E). Purifying selection was more likely for CPA1, CPA3, and CPO, although the sample size for non-CPO genes was small. When combined and compared with CPO genes (Fig. 5F), the data suggested statistically significant purifying selection acting upon CPO duplicates, with less stringent selection or neutral selection acting upon other CPA/B gene duplicates. This was also reflected in the p values reported for this test of purifying selection (Fig. 5G). CPO paralogs suggested a bimodal distribution, with one group of values for dS—dN in the range of 1 to 4.5, and another in the range of 7 to 14 (see Fig. 5F). Interestingly, most of the low values involved genes predicted to be either pseudogenes or pseudoenzymes, which might be expected if selective pressures are not acting upon such genes.

### Xenopus CPO paralogs show evidence of neofunctionalization

The data thus far suggests that CPO genes are frequently duplicated and maintained in fish and *Xenopus* genomes, and that these duplicates are maintained through purifying selection, suggesting functional importance. In many cases (~ 30% of duplicated CPO genes; see Fig. 5d), the specificity-determining residue has changed from the stereotypical arginine, found in most CPO proteins, to a hydrophobic or polar residue, suggesting a new function that could be selected for. In order to experimentally examine such predictions, we obtained cDNAs for the four *Xenopus tropicalis* CPO paralogs, engineering each of these cDNAs to include an HA tag for protein detection (see Supplementary Methods online for sequence details).

A number of characteristics could be determined simply from the amino acid sequences of these four predicted proteins, using available prediction programs. In contrast to most fish and mammalian CPO orthologs which lack a complete prodomain, these four *Xenopus* CPO orthologs retained a prodomain (see Supplementary Fig. S6 online for multiple alignment), although only Cpo.1 was predicted by ProP 1.0 to be cleaved by a proprotein convertase at the junction between the prodomain and the CP domain (ProP 1.0; https://services.healthtech.dtu.dk/service.php?ProP-1.0)^34^. One of the four, Cpo.2, was not predicted to encode a GPI signal peptide (PredGPI; http://gpcr.biocomp.unibo.it/predgpi/)^35^, although all were predicted to encode an ER signal peptide (SignalP-5.0; https://services.healthtech.dtu.dk/service.php?SignalP-5.0)^36^. Only Cpo.1 encodes an arginine at the substrate specificity-determining position. Based on this site alone, the other CPO paralogs would be predicted to exhibit differing substrate specificity: a glycine is found at this position in Cpo.2, a glutamine in Cpo.3, and a cysteine in Cpo.4. To our knowledge, the consequence of these amino acids in determining substrate specificity has not been previously investigated experimentally. A phylogenetic analysis of these four proteins (see Supplementary Fig. S1 online) suggests that Cpo.3 was the progenitor gene, duplicating to produce Cpo.1 (which then freed Cpo.3 to adopt an alternate substrate specificity), which subsequently duplicated to produce Cpo.2 and Cpo.4. This is consistent with gene synteny shown in Fig. 3.

**Figure 6:**
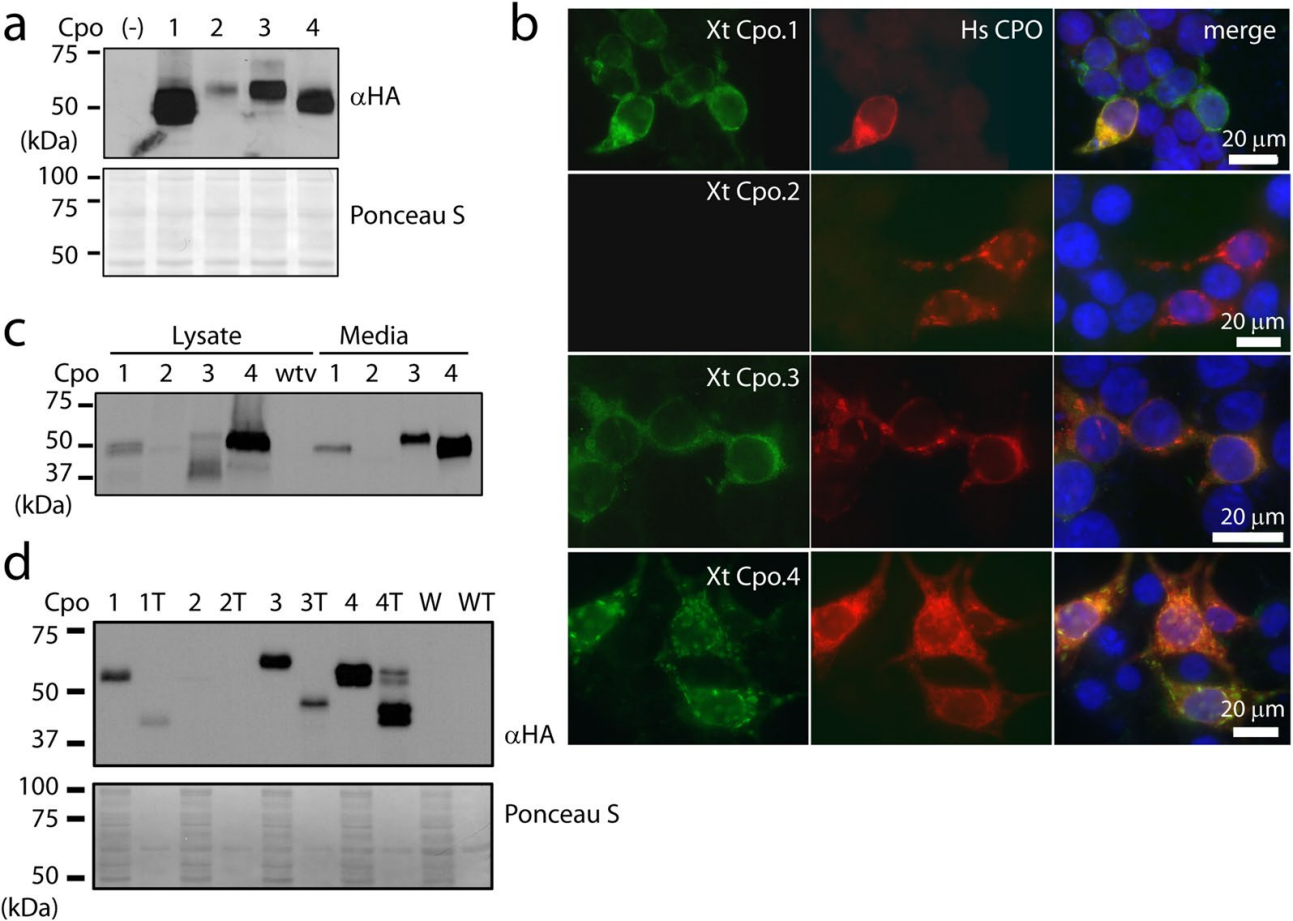
Xenopus tropicalis CPO orthologs are expressed and processed by an endopeptidase. (**a**) HEK293T cells were transfected with plasmids encoding the four HA-tagged X.t. CPO orthologs, or an empty plasmid (−). Cell lysates (equal amounts of protein) were resolved by SDS-PAGE and western blotted with an anti-HA antibody. The nitrocellulose membrane was also stained with Ponceau S as a loading control. (**b**) The distribution of HA-tagged X.t. CPO orthologs (green, HA antibody) was analyzed in transfected HEK293T cells by immunocytochemistry and compared with the distribution of transfected human CPO (red, CPO antibody). (**c**) The four X.t. CPO orthologs were expressed in Sf9 cells using recombinant baculoviruses. Cells were infected with wild-type virus (wtv) as a control. One percent of each cell lysate, or 0.06% of each collected medium, was resolved by SDS-PAGE and western blotted using an HA antibody. (**d**) Sf9 conditioned media (containing cpo.1, 2, 3, or 4, or wild-type virus (W)) and the same media incubated with 2.5 µg/ml trypsin (T) for 5 min at room temperature were resolved by SDS-PAGE and western blotted with an HA antibody. The membrane was stained with Ponceau S as a loading control.

Expression plasmids encoding HA-tagged *Xenopus* CPO paralogs were transfected into HEK293T cells and protein expression analyzed by western blotting. All four CPO paralogs were detected in cell extracts, with Cpo.1 expressed most highly and expression of Cpo.2 being the weakest (Fig. 6A). No expression was detected in media, and no enzymatic activity could be detected from either media or cell extracts, even following limited digestion with trypsin.

Immunocytochemistry was employed to determine the subcellular distribution of these Xenopus CPO paralogs in comparison to that of human CPO in HEK293T cells. Expression of Xenopus paralogs partially overlapped that of human CPO in a punctate pattern (Fig. 6B) consistent with that observed previously^37^. Xenopus Cpo.2 was not detected.

As enzymatic activity was not detected when Xenopus CPO paralogs were expressed in the HEK293T system, we used the Sf9 insect cell system for more robust expression. Infection of these cells with baculovirus encoding Xenopus CPO paralogs resulted in expression of all four paralogs, as detected by western blot, both in the cell lysates and in the conditioned medium (Fig. 6C). Although band intensity appeared similar in both cell lysates and media, these bands resulted from the analysis of 1% of the total cell lysate and 0.06% of the total media collected, indicating the majority of the protein to be secreted into the media. Highest expression was detected for Cpo.4, followed by Cpo.3 and Cpo.1. Expression of Cpo.2 was at the detection limit of our western blotting system. As it was likely that the prodomains found on these CPO paralogs would be cleaved off in their natural environment, conditioned media were incubated with small amounts of trypsin at room temperature for five minutes, followed by inactivation of trypsin with PMSF. Western blotting showed that this cleavage was effective in producing mature enzymes, with molecular weights similar to those predicted for the mature enzymes, with the exception of the weakly-expressed Cpo.2 (Fig. 6D).

**Figure 7:**
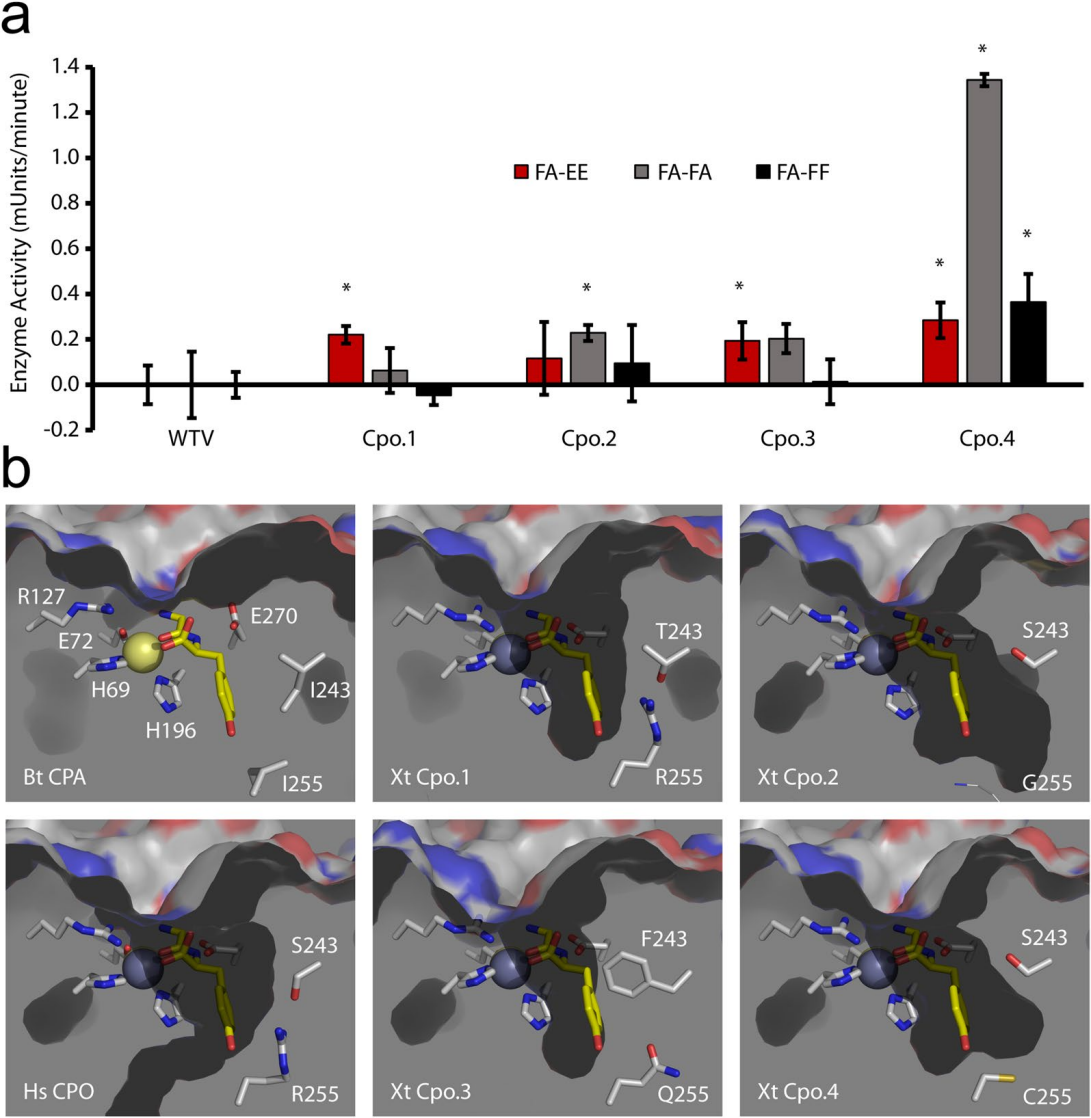
Xenopus tropicalis CPO orthologs exhibit different substrate preferences. (**a**) One hundred microliters of each trypsinized media was incubated with 900 µl of 0.5 mM substrate (FA-EE, FA-FA, FA-FF, pH 7.5) at room temperature. Change in absorbance at 340 nm was measured over time and the rate of reaction shown as the change in absorbance (milli-absorbance-units) per minute. n = 3–6. Error bars indicate standard deviation. *p < 0.05, comparing to the corresponding WTV dataset, as determined by ANOVA and Tukey–Kramer post-hoc analysis. (**b**) Each Xenopus tropicalis CPO paralog was modeled with AlphaFold2 and aligned in Pymol with X-ray crystal structures for Bos taurus CPA (3CPA) and Homo sapiens CPO (5MRV, chain a). All images show the zinc cofactor from Hs CPO as a gray sphere, the zinc cofactor from Bt CPA as a yellow sphere (largely superimposed by the gray sphere) and the Gly-Tyr dipeptide bound to the active site of Bt CPA as a yellow stick model. Key active site residues from each structure are indicated. Cavity surfaces, as viewed from the inside of the protein and with the prodomains removed, are shown in dark grays, while the protein outer surface is shown with white carbons, red oxygens, and blue nitrogens. No substrate binding pocket is shown for Bt CPA, as the pocket is filled with the Gly-Tyr and so not rendered as a surface.

Enzyme assays confirmed the above activation of CPO paralogs by tryptic removal of their prodomains—while no enzyme activity could be detected in conditioned medium, trypsinized medium showed robust activity for Cpo.4 and weaker activity for Cpo.1 and Cpo.3, consistent with the lesser expression levels of Cpo.1 and Cpo.3 (Fig. 7A). Most interesting was a comparison of substrate specificity, utilizing the three synthetic 3-(2-furyl)acryloyl-amino acid substrates available, where the FA chemical group is conjugated to a dipeptide (Glu-Glu (EE), Phe-Ala (FA), or Phe-Phe (FF)). Cpo.1 was able to cleave FA-EE, but not FA-FA or FA-FF, consistent with the presence of an arginine at position 255. Cpo.2, although not detected in large quantities by western blotting, was able to cleave FA-FA, but a statistically significant cleavage of the other two substrates was not detected. Cpo.3 appeared equally able to cleave FA-EE and FA-FA, although the *p* value for activity against FA-FA was 0.09, and thus not statistically significant in this analysis, while Cpo.4 exhibited a clear preference for FA-FA over the other two substrates.

To examine the structural basis of the unique substrate specificities observed for the four Xenopus CPO paralogs, AlphaFold2 models were prepared (Fig. 7B). Previous work has indicated that, while position 255 is key in determining specificity^24^, additional positions also contribute to the shape and size of this binding pocket, including positions 203, 207, 243, 247, 253, and 268^38^ (see residues highlighted gray in Supplementary Fig. S6 online). While most of these positions remained identical across all Xenopus CPO paralogs, position 243 contained a phenylalanine in Cpo.3 in contrast to the serine or threonine found in other paralogs, resulting in a much narrower pocket than other models (Fig. 7B). This was consistent with the inability of this enzyme to cleave C-terminal phenylalanine (Fig. 7A). Because Cpo.3 could cleave C-terminal glutamate in a manner similar to Cpo.1, it seems likely that Q255 provides enough electrostatic potential for interaction with the charged glutamate. Cpo.2, Cpo.3, and Cpo.4 all preferred C-terminal alanine over phenylalanine. This could be due to the shape of the binding pockets of these enzymes, which were slightly narrower at the top, providing sufficient contacts with the short alanine side chain, yet quite wide at the bottom, perhaps providing too much space to make sufficient contacts with the larger phenylalanine side chain.

## Discussion

Gene duplication is a random event that occurs during the history of any species. It likely occurs at a constant rate, with some dependence on factors such as amount of repetitive sequence in a genome and the presence of transposon-like elements. Following any duplication event, each resulting gene will be subjected to selective forces, both negative and positive, depending on gene function and impact on organismal fitness.

Here we have investigated recent duplications identifiable in the M14 family of MCPs. We have found four of the 23 M14 MCP genes to be frequently duplicated within vertebrate genomes. Three of these genes, AEBP1, CPXM1 and CPZ, were duplicated once, likely through a whole-genome duplication event in the teleost fish lineage, while CPO has been tandemly duplicated many times in fish and Xenopus lineages. Time tree analysis using one calibration point at the divergence of Xenopus frogs and ray-finned fishes (see Supplemental Fig. S2 online) suggested that all three Xenopus duplication events occurred well before the divergence of the X. tropicalis and X. laevis progenitor and subsequent allotetraploid fusion events suggested to be 34 and 17 million years ago (Ma), respectively^39^. This was consistent with the presence of these four paralogs in both Xenopus species. Time tree analysis also suggested that most duplication events within fish lineages occurred during the Cretaceous and Cenozoic eras, consistent with the great diversification of bony fishes during this time^40^.

Our analyses suggest that both functional advantages provided by duplicated genes and intrinsic features of the genes themselves lead to duplication and retention. For example, we show that the sizes of duplicated MCP genes tend to be smaller, increasing chances of complete duplication and decreasing chances for sequence degradation. This effect of gene size has been previously reported in several studies. A study of Brassicaceae genomes showed that genes transposed as complete copies were significantly shorter than genes transposed as truncated copies^41^. A study of the maize genome showed that tandem duplicates were smaller than all other genes^42^. A broad analysis of the human and mouse genomes has also found this to hold true^43^. Our study supports these broader studies with a careful analysis of one gene family.

Gene size notwithstanding, any gene that is to be conserved in duplicate must have a function that contributes to fitness when doubly expressed. To determine if any particular functions were overrepresented in duplicated genes within prokaryotes, Bratlie et al. examined the GO terms of duplicated genes compared with those of singletons^44^. They found that genes producing factors that may be involved in environmental adaptation were overrepresented in the duplicate dataset. A similar proposal was made by Zhang in 2003, who suggested that genes involved in immunity, reproduction and sensory systems probably have high rates of gene birth and death^45^. Plant studies have suggested that the mode of gene duplication matters, due to dosage effects: transcription factors, protein kinases, and ribosomal proteins are retained after whole-genome duplication events^46,47^, while stress response genes are preferentially retained after local duplications^48^.

Are there any functional similarities in our small dataset of four duplicated MCP genes that differentiate them from other MCPs? All of the four function primarily in the extracellular space: CPO is a GPI-anchored enzyme that will process extracellular factors, while the other three are found in the extracellular matrix^30,49–51^. It has been suggested that matrix processing enzyme genes are overrepresented in vertebrate genomes^52^. It could be that the extracellular matrix presents a less restrictive environment, flexible and accommodating to different complements of protein. In many cases, the extracellular matrix is the first place of contact with the environment, where flexibility for adaptation is necessary. In fact, the extracellular matrix is uniquely expanded in multicellular eukaryotes due to its many diverse roles and needs for innovation^53^.

Another feature that may connect these four genes relates to their functions. Two of the four, AEBP1 and CPXM1, are pseudoenzymes that likely function through interactions with other extracellular matrix proteins^29,30,54,55^. CPZ is known to have enzymatic activity, but also contains a frizzled domain with an important role in protein interaction^56^. None of these functions require the removal of a prodomain from these proteins, suggesting that duplication of these genes could result in immediate function that could be selected for. In contrast, a protein requiring activation through prodomain removal could be deemed useless if expressed in a location lacking the required processing enzyme, leading to subsequent pseudogenization.

A number of roles have been proposed for CPO, including digestion of dietary proteins and peptides^24,50^, modification of extracellular growth factors and bioactive peptides^24^, and control of cellular lipid droplet quantity^37^. Whatever the primary role for CPO is, it seems apparent that it is non-essential, as CPO is a pseudogene in a large set of rodent genomes (Fig. 2)^25^. This presents an interesting situation in which the CPO gene is a nonfunctional pseudogene in many organisms, yet provides sufficient contribution to fitness to be duplicated, often many times, in other organisms. This conundrum can be resolved by considering a comparison of essential and non-essential genes in *C. elegans* where it was shown that non-essential genes are more likely to be successfully duplicated and more likely to be lost as well^57^. Two models were proposed to account for this, with evidence supporting both: a mutational model in which non-essential genes are present in chromosomal regions with high recombination rates, and a selectional model, in which non-essential genes do not exhibit the dosage sensitivity found for essential genes (due to the functions of essential gene products within complexes). In the case of Xenopus CPO paralogs, we found evidence of a nearby transposase-like gene and a large cluster of crystallin genes, suggesting a chromosomal region amenable to recombination. However, the apparent simplicity of CPO, lacking a prodomain in most cases and with a restricted expression profile, suggests that the selectional model could apply as well.

The CPO protein has been shown to have enzymatic activity^24,37,50^. In contrast to closely related enzymes, most orthologs of the CPO enzyme do not include a prodomain^50^, possibly predisposing this gene to immediate function upon duplication and the possibility for neofunctionalization. We show that following gene duplication in Xenopus, the resulting four CPO genes have undergone mutations leading to changes in substrate preference that likely have played a role in their fixation in the population. While CPO1 retains the acidic C-terminal amino acid preference found in other CPO enzymes, CPO3 shows no preference between C-terminal glutamate or alanine, and CPO4 shows a clear preference for the cleavage of C-terminal alanine. This data is also the first time, to our knowledge, that the substrate specificity implications of polar residues at the base of the specificity pocket in position 255 have been explored. A glutamine at position 255 in CPO3 results in no preference between acidic or nonpolar residues, while a cysteine at this position in CPO4 also allows for a broad range of substrate binding. Our modeling suggests that the overall size and shape of the substrate binding pocket, in addition to the identity of residue 255, is responsible for the fine-tuning of substrate specificity. It might be noted that CPO2 exhibited weak activity with substrate preference similar to that of CPO4, although little to no expression was detected. This could indicate a C-terminal cleavage resulting in the removal of the HA-tag. Further analysis following purification would be necessary to clarify this possibility.

## Materials and methods

### Bioinformatics data sources

Vertebrate orthologs of all human M14 metallocarboxypeptidases were identified within the Ensembl genome database (release 98). This release included genomes for 92 mammals, 34 birds and reptiles, and 62 fish. Ensembl also provided the size for each gene, as measured from the beginning of the first exon to the end of the last exon. Gene synteny information was acquired from the Ensembl database (release 100) and its related resource, Genomicus. RNAseq gene expression data was acquired from proteinatlas.org. Consensus normalized expression (NX) levels for 55 tissue types was used, created by combining the data from three transcriptomics datasets (HPA, GTEx and FANTOM5) using an internal normalization pipeline.

### Bioinformatics analyses

Multiple alignments were created using Clustal Omega and presented using Jalview. Phylogenetic trees were created with IQ-TREE 2^58^. Branches were tested by SH-like aLRT with 1000 replicates as well as 1000 ultrafast bootstrap iterations. The best-fit model used for phylogenetic reconstruction of the MCP family (Fig. 1A) was LG + I + G4. Phylogenetic trees were prepared for presentation in Dendroscope^59^. Time trees were prepared using the RelTime-ML feature in MEGA-X.

### Protein structure modeling

Models were prepared using AlphaFold2 through ColabFold, using MMseqs2 to generate sequence alignments. Best models were rendered in Pymol.

### Plasmids

Xenopus CPO paralog cDNAs were obtained from Twist Bioscience in the pTwist CMV WPRE Neo plasmid. All cDNAs were modified with an HA tag either at the C-terminus of the protein sequence (Cpo.2) or immediately before the GPI signal sequence (Cpo.1, Cpo.3, Cpo.4; see sequence details in Supplementary Methods online). Coding sequences were cut out of the pTwist plasmids using NotI and XbaI restriction enzymes and ligated into the same restriction enzyme sites in pVL1392 using standard cloning methods. Plasmids were transformed into DH5α cells using standard methods and purified using the nonionic detergent (NID) method^60^.

### Mammalian cell culture and transfection

HEK293T cells were grown at 37 °C with 5% carbon dioxide and maintained in Dulbecco’s Modified Eagle Medium that contained 10% fetal bovine serum (Sigma Aldrich) and 1% penicillin–streptomycin. For transfections, 1.5 × 10^6^ HEK293T cells were seeded on a 60 mm dish in growth medium 24 h before transfection. Five hundred microliters of serum-free DMEM were mixed with 5 μg of plasmid DNA and 15 μl of 1 µg/µl polyethyleneimine (PEI; 25 kD linear from Polysciences). After incubation at room temperature for 15 min, the mixtures were added to the cells.

### Sf9 insect cell culture and viral infection

Sf9 cells were grown in Sf900 II serum-free medium (ThermoFisher) at 28 °C with shaking at 140 rpm. In preparation for transfection for baculoviral particle assembly, 1 × 10^6^ Sf9 cells were plated in each well of a 6-well plate. ProGreen linearized baculovirus DNA (AB Vector) was co-transfected with pVL1392 plasmid and ProFectin transfection reagent (AB Vector) according to the manufacturer’s instructions. Following two rounds of viral amplification, the viral stock was used to infect Sf9 cells in suspension at 2 × 10^6^ cells/ml and 2% virus stock.

### Protein extraction and western blotting

Cellular protein extracts were collected in cold lysis buffer containing 150 mM NaCl, 20 mM Tris pH 7.5, and 1% Nonidet P-40. Protease inhibitors (Cocktail III, Research Products International) were often added, unless subsequent tryptic proteolysis was to be performed. Following passage through a 25GA needle, extracts were centrifuged to pellet cell debris. In many cases, conditioned growth medium was also collected and centrifuged to remove any debris.

Proteins were resolved by SDS-PAGE on 10% gels and transferred to nitrocellulose. Western blotting was performed according to standard protocol with mouse HA-tag (6E2) primary antibody (Cell Signaling Technology; 1:2000 dilution) and horseradish peroxidase-conjugated secondary antibody (Cell Signaling Technology, 1:2000 dilution). BioRad Precision Plus Protein Dual Color Standards were used as protein markers for all protein gels. Images were obtained using LumiGLO chemiluminescent reagent (Cell Signaling Technology).

### Enzyme assays

One hundred microliters of cell extract or media were mixed with 900 ul of 0.5 mM substrate (3-(2-furyl)acryloyl-amino acids, also known as FA-peptides, specifically FA-EE, FA-FA, or FA-FF; Bachem) dissolved in 50 mM Tris pH 7.5 and 150 mM NaCl. Absorbance at 340 nm was measured every 5 s for 5 min at room temperature (23 °C). Enzyme activity was reported as the decrease in absorbance (mUnits) per minute.

### Immunocytochemistry

HEK293T cells were grown and transfected on poly-D-lysine-coated coverslips. Cells were fixed with 4% paraformaldehyde in phosphate-buffered saline (PBS) for 10 min at room temperature. They were then washed with PBS 3 times for 5 min each and permeabilized with 0.1% Triton X-100 in PBS for 15 min. Following three five-minute washes in PBS, cells were blocked with 5% bovine serum albumin (BSA) for 45 min. Incubation with primary antibodies (RP3-CPO from Triple Point Biologics, 1:1000 dilution; mouse HA-tag (6E2) antibodies from Cell signaling Technology, 1:1000 dilution) diluted in 5% BSA in PBS was performed for one hour at room temperature. After three five-minute washes in PBS, secondary antibodies (anti-rabbit Alexafluor-555 and anti-mouse Alexafluor-488 from Cell signaling technology, 1:1000 dilution) were added and incubated for one hour. Following three five-minute washes in PBS, coverslips were inverted on 8 μl of aqueous mounting medium (PBS containing 1 mg/ml *p*-phenylenediamine and 1 μg/ml DAPI) and sealed with nail polish. Imaging was performed on a Keyence BZ-X810 fluorescence microscope.

### Statistical analysis

The non-parametric Kruskal–Wallis statistical test was performed to analyze the relationship between gene copy number and gene size or gene expression. Enzyme activity data was compared through ANOVA with Tukey–Kramer post-hoc test.

## Supplementary Information

Supplementary Information 1.

Supplementary Information 2.

## Data Availability

All data generated or analyzed during this study are included in this published article and its Supplementary Information files, or are publicly available at www.ensembl.org.
