# Supplementary material for: Acquisition of new function through gene duplication in the metallocarboxypeptidase family
Source: Sci Rep. 2023 Feb 13;13:2512. doi: 10.1038/s41598-023-29800-9 (PMC9925722; doi:10.1038/s41598-023-29800-9)
Supplement: Supplementary file 1 — Supplementary Information 1. [file 41598_2023_29800_MOESM1_ESM.pdf]

Supplementary Information

Acquisition of new function through gene duplication in the metallocarboxypeptidase family

Daniel Fajardo, Ritchie Saint Jean, and Peter J. Lyons

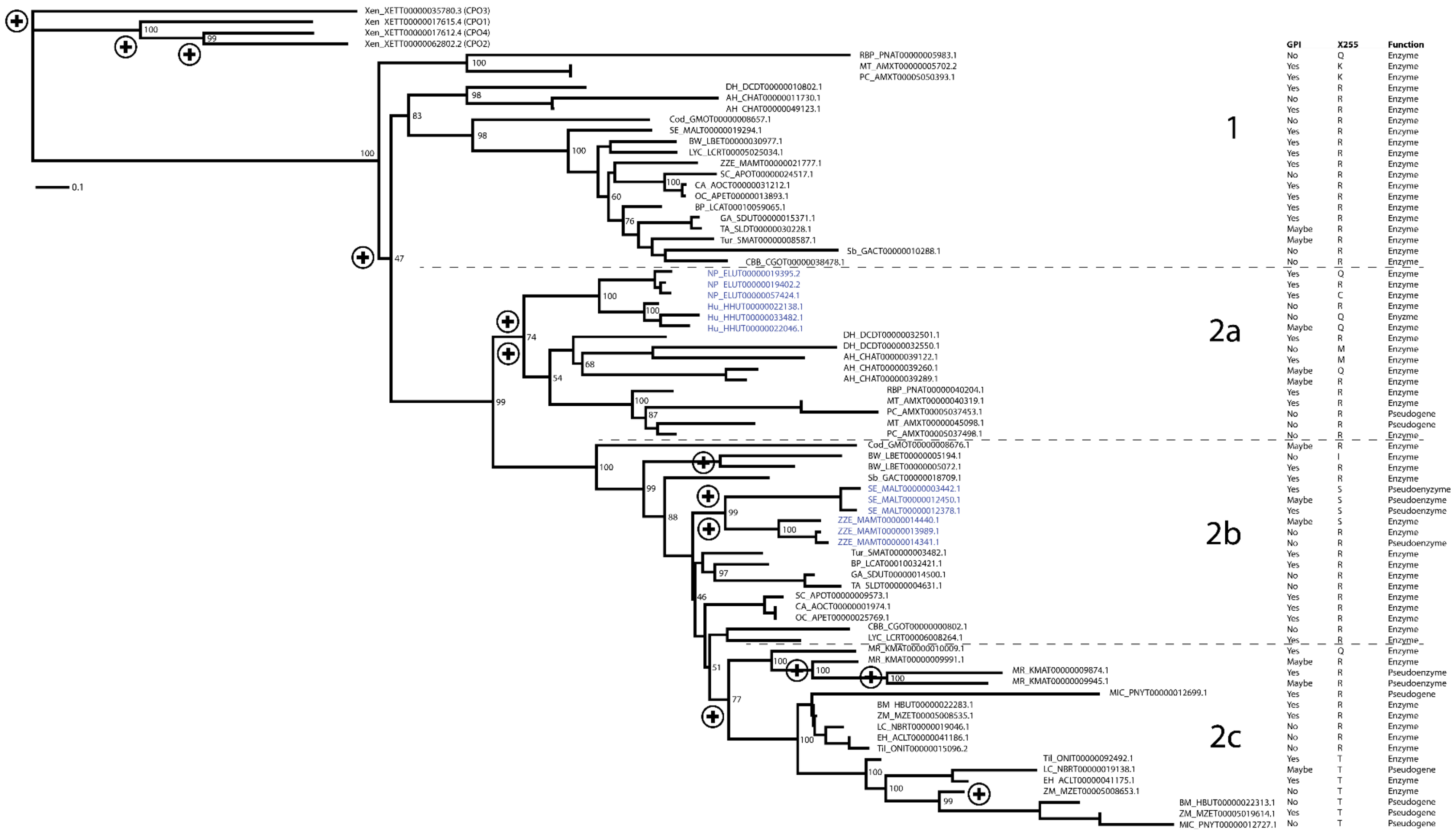

Supplementary Figure S1. A phylogenetic tree showing relationships among all duplicated CPO genes from fish and *Xenopus tropicalis*. Tree was created by IQTREE2 using indicated Ensembl protein sequence data; branches were tested by SH-like aLRT with 1000 replicates, as well as 1000 ultrafast bootstrap iterations. All apparent gene duplication events are marked by a ⊕. These appear to result in two major groups within the fishes (groups 1 and 2), with group 2 genes subsequently undergoing further duplication events resulting in groups 2a, 2b and 2c. Predictions of GPI anchoring, substrate specificity, and function are listed to the right.

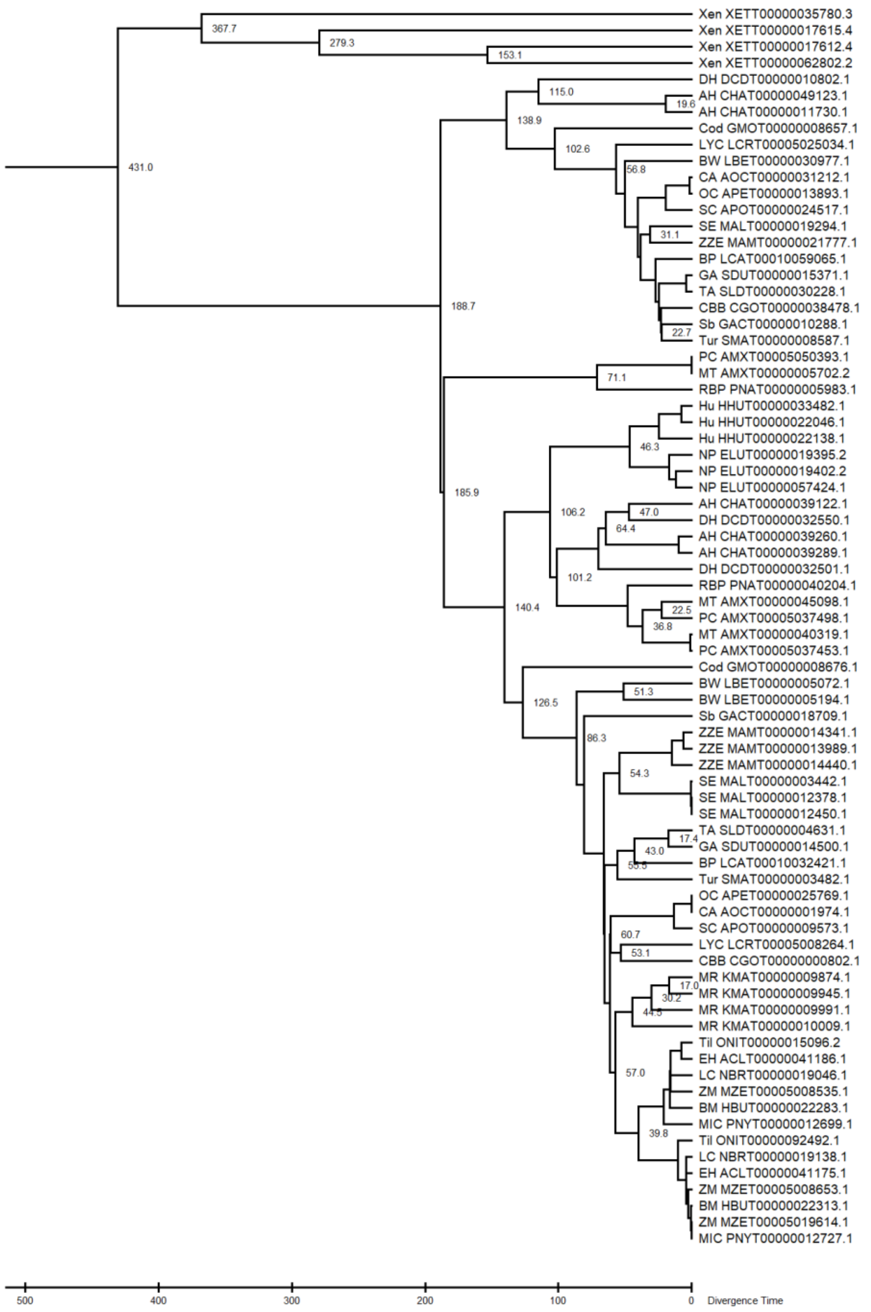

Supplementary Figure S2. A time tree showing estimated divergence times for CPO duplication. Divergence times were calculated using the RelTime-ML approach in MEGA-X, using a Clustal Omega alignment and a phylogenetic tree constructed as in Supplementary Figure S1, with the addition of the Xenopus tropicalis CPA6 amino acid sequence (ENSXETT00000123092.1) as the outgroup. The divergence of Xenopus frogs and Actinopteri fish sequences was calibrated at 431 Ma with a 95% CI of 422-440 Ma, based on a median time from 33 studies curated at timetree.org.

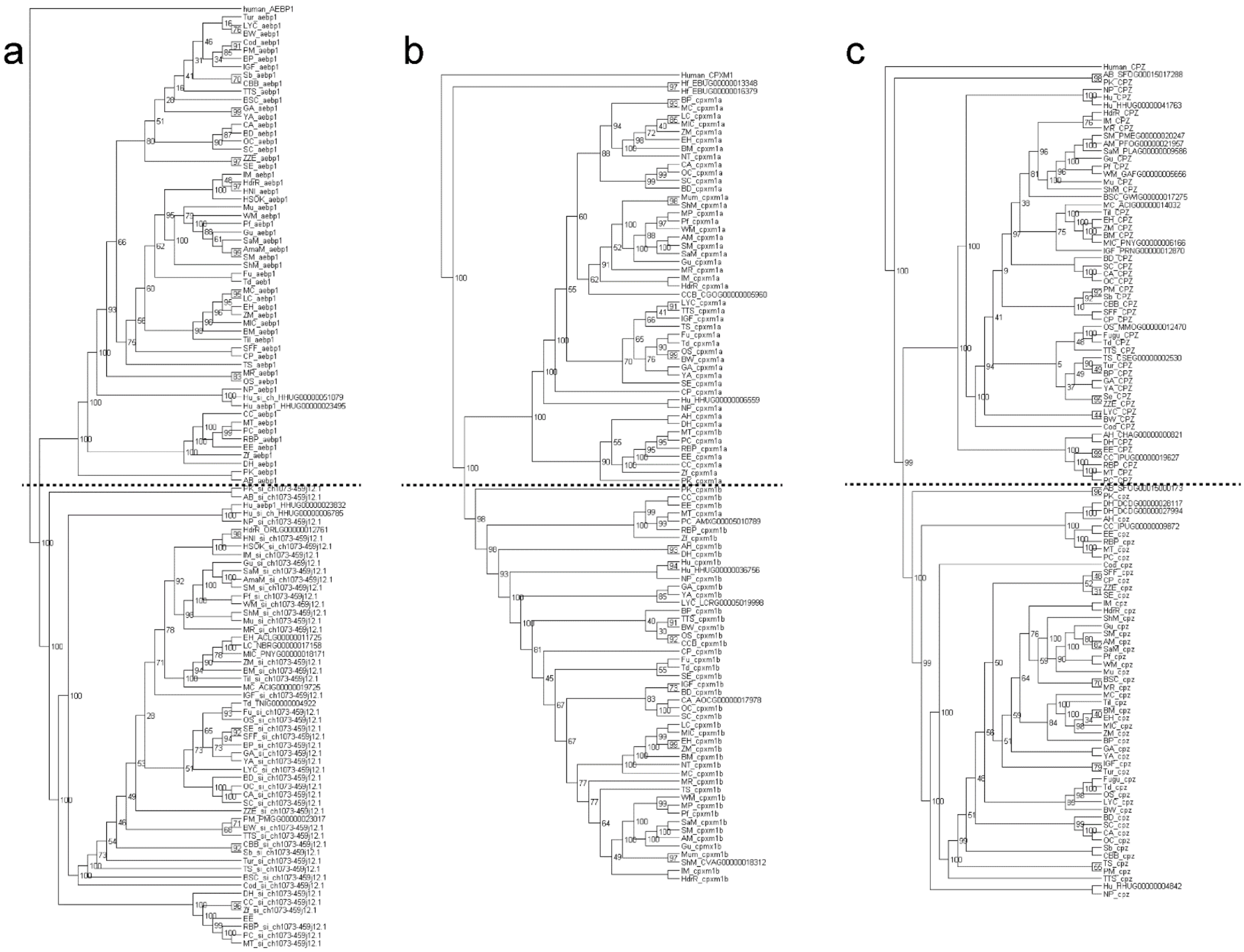

Supplementary Figure S3. Phylogenetic trees showing relationships among all AEBP1, CPXM1 and CPZ gene duplicates.

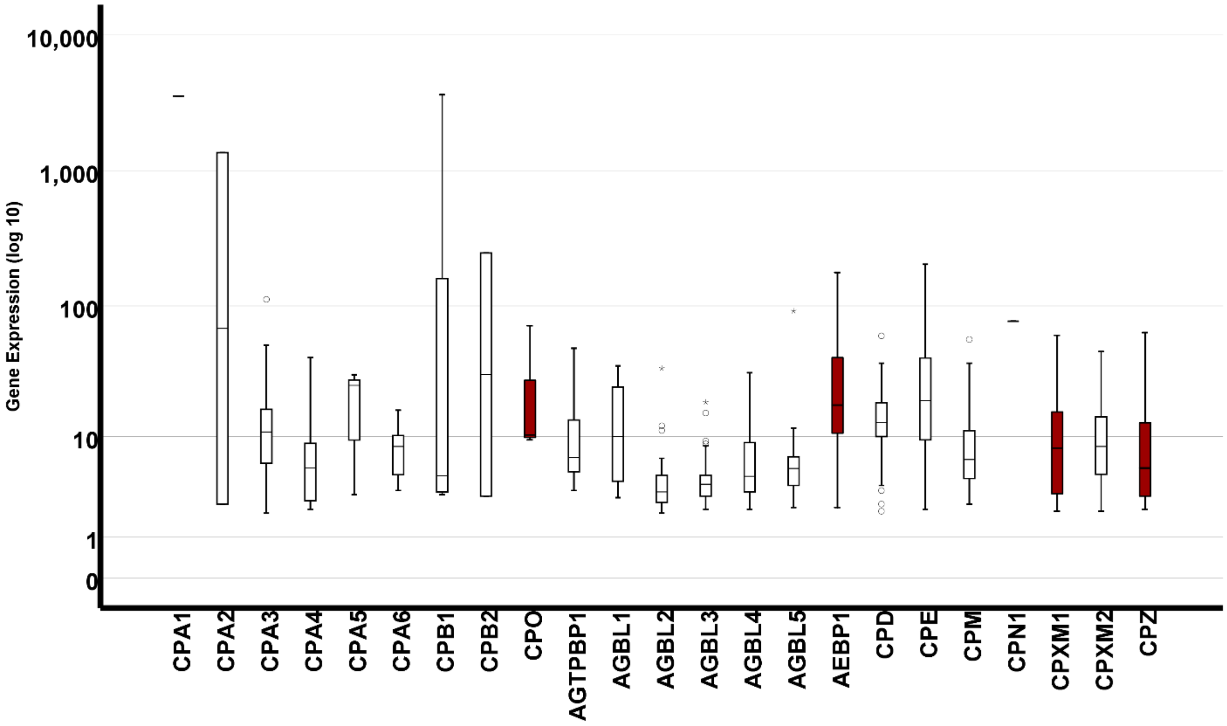

Supplementary Figure S4. Gene expression levels do not impact rates of gene duplication within the CP family. Expression levels for the indicated human genes were curated from proteinatlas.org. Expression levels of all tissues were averaged for each gene. An independent sample t-test was performed comparing all frequently duplicated genes (red) to all others (white; p= 0.657).

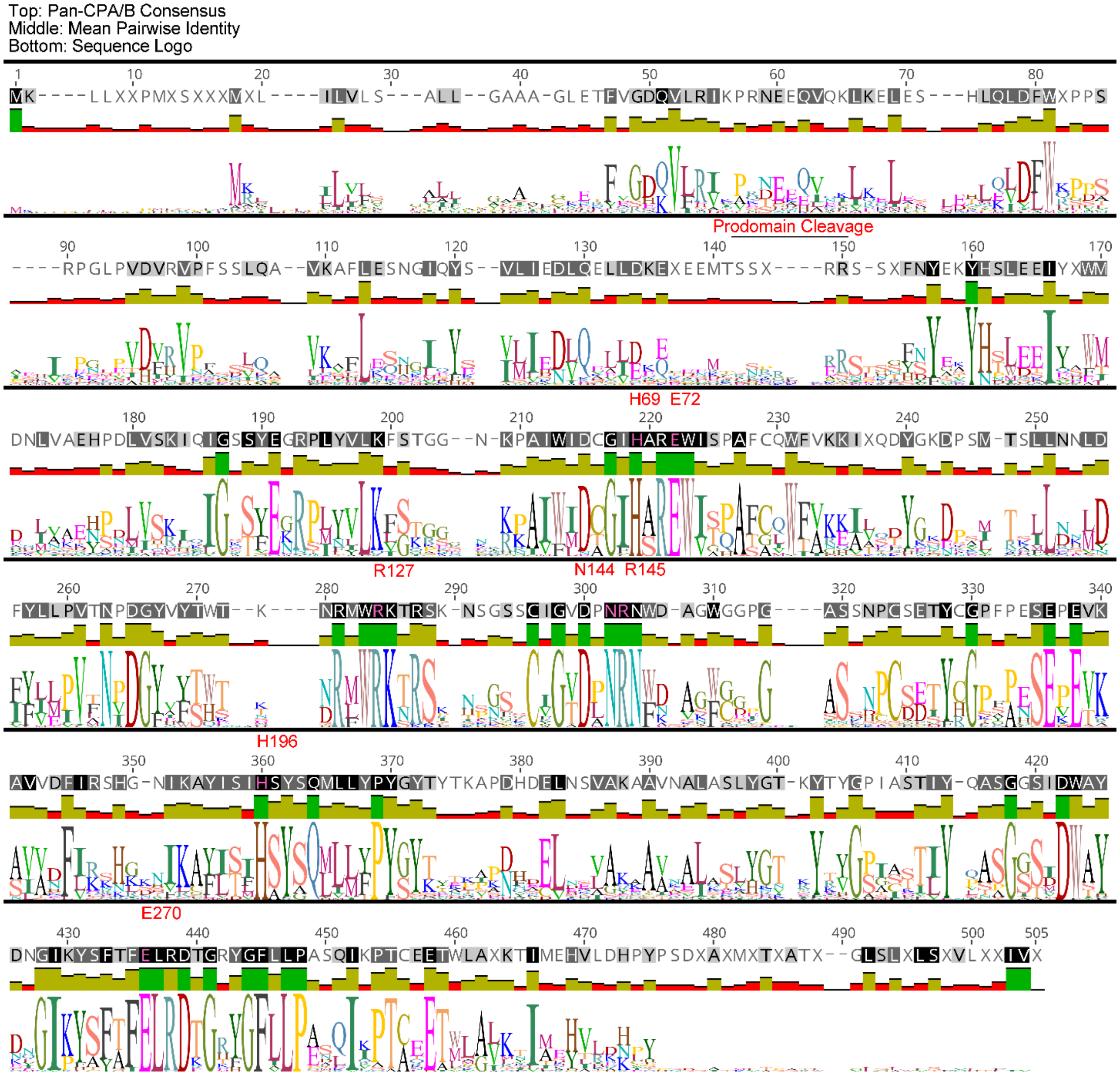

Supplementary Figure S5. In order to categorize predicted proteins as pseudogenes, pseudoenzymes, or active enzymes, a multiple alignment was created using Clustal Omega, consisting of a broad range of vertebrate carboxypeptidase sequence representatives (CPA1, CPA2, CPA3, CPA4, CPA5, CPA6, CPB1, CPB2, and CPO) typically including five mammal sequences (including one afrotherian, one marsupial, and one monotreme), four sauropsid sequences (two bird and two reptile), one amphibian sequence, and two fish sequences. Key catalytic residues are indicated in red, numbered as in bovine CPA, as is the convention in this field.

Supplementary Figure S6. A multiple alignment of amino acid sequences for human CPO, Xenopus tropicalis Cpo.1, Cpo.2, Cpo.3, Cpo.4, and bovine CPA1. Predicted ER signal peptides are shown in blue, predicted prodomains are shown in red, enzymatic domains are shown in black, and predicted GPI signal peptides are shown in green. Key active site residues are highlighted and numbered below by their position in bovine CPA1, the convention in this field.

```
predicted ER signal peptide; predicted prodomain; enzymatic domain;
predicted GPI signal peptide; key active site residues; additional substrate
specificity-determining residues

hCPO: human CPO
xCpo: Xenopus CPO
bCPA1: Bovine CPA1


                    10         20         30         40         50         60
hCPO        MKPLLETLYL LGMLVPGGLG ---------- ---------- ---------- ----------
xCpo.1      MKRILLCICI LGILTQVGAY LKVQYNGDQV LKLTLKTTDD CEHMQNI--C KQLQLDLWKP
xCpo.2      MIFLLGTIFL LGVQVYEGSC LTVQYNGDNV FKITPETSEH AQYLQTL--A NEWLLDLWRP
xCpo.3      -MHHLWIVWT FGTLTLQVCC TGTEYDGGNI LEITPESEKQ VQCLQNI--L QSWLLDLLKP
xCpo.4      MKLFNLWFCL LGILVYEGFC MKVKYDGDQV LKMIPQTLKH AQFMQGL--I QEWMLDLWKP
bCPA1       MQGLLILSVL LGAALG---- -KEDFVGHQV LRITAADEAE VQTVKELEDL EHLQLDFWRG

                    70         80         90        100        110        120
hCPO        ---------- ---------- ---------- ---------Y DRSLAQHRQE I--VDKSVSP
xCpo.1      SRIEDIQPGK EMHVRIPFPL LQKFKENLHQ YSIPFEVMIK DVQKLIDSSN V--GDYRRQK
xCpo.2      QTVEQIHEGS DIHVQIPFAY MEQMKQKLLQ HSIPYEVLIN DVQKLIDSNT V--SAPKIQK
xCpo.3      LQPEDINVKT TVHVRIPSTA LQLVKEDLLH CSQSLEILTG NVKYIEE--D K--IDTKETR
xCpo.4      VMVEQIQAGR EMHVRVPFSH LQEIKEKLLQ NMLPYQILIS DVQELVNRNT P--IETKMQK
bCPA1       P----GQPGS PIDVRVPFPS LQAVKVFLEA HGIRYRIMIE DVQSLLDEEQ EQMFASQSRA

                   130        140        150        160        170        180
hCPO        WSLETYSYNI YHPMGEIYEW MREISEKYKE VVTQHFLGVT YETHPMYYLK ISQPSGNPKK
xCpo.1      KILAEFDYTT YHPMDEIYQW MDQVKEAYSD LVSMHYLGST YELRPIYYFK IGWPSDKQKK
xCpo.2      ASLENYDYTK YHPMDEIYNW MDLMKEKHSE IVSQHYIGCT YELRPMYYLK IGWPSDKQKK
xCpo.3      KTINEYNYTT YHPMNEIYDW INGIAKKHSQ FVTQHLLGLT YESRPMQYLK ISQPSENHKK
xCpo.4      ISLDNYDYTK YHPMDEIYDW MEQIQLKHRD LVTKHFMGST YELRPIYYFK IGWPSDKPKK
bCPA1       RSTNTFNYAT YHTLDEIYDF MDLLVAEHPQ LVSKLQIGRS YEGRPIYVLK FSTGG-SNRP

                   190        200        210        220        230        240
hCPO        IIWMDCGIHA REWIAPAFCQ WFVKEILQNH KDNSSIRKLL RNLDFYVLPV LNIDGYIYTW
xCpo.1      IIWMDCGIHA REWIAVAYCQ WFVKEILETH KTNPLLQKVL HNIDFYIVPV LNIDGFVYSW
xCpo.2      IFFIDCGFHA REWISVAFCQ WFVNEIVSHY KTDAILANVL KQVDFYVLPV MNIDGYVYTW
xCpo.3      IVWIDCGIHA REWIAPAFCQ WFVKEIVQNY QNDQRIRKIL QNLDIYVLPV LNIDGYIYSW
xCpo.4      IIFMDCGIHA REWIAVAYCQ WFVKEILSSH SNNKLLTNVL KQVDFYVVPV FNIDGYIYSW
bCPA1       AIWIDLGIHS REWITQATGV WFAKKFTEDY GQDPSFTAIL DSMDIFLEIV TNPDGFAFTH
                    H69 E72
```

```
                           250        260        270        280        290        300
hCPO                TTDRLWRKSR SPHNNGTCFG TDLNRNFNAS WCSIGASRNC QDQTFCGTGP VSEPETKAVA
xCpo.1              NVNRLWRKSR SPHNNGSCYG VDLNRNFNSK WGSIGASNNC RDETYCGTGP ASEPEVNAVS
xCpo.2              TTNRLWRKNR SPHENGTCYG VDLNRNFDSQ WCSIGASRDC NSNTFCGPEA ASEPETKALS
xCpo.3              TKERLWRKNR SQYGNGTCYG VDLNRNFNVS WCTHRSSTNC SSNSFCGSSP VSEPETRAVV
xCpo.4              TTERLWRKNR SPHNNATCYG VDLNRNFNSS WCSVGASRDC NSQTFCGSAP ASEPETQAVA
bCPA1               SQNRLWRKTR SVTSSSLCVG VDANRNWDAG FGKAGASSSP CSETYHGKYA NSEVEVKSIV
                          R127                 N144R145

                           310        320        330        340        350        360
hCPO                SFIESKKDDI LCFLTMHSYG QLILTPYGYT KNKSSNHPEM IQVGQKAANA LKAKYGTNYR
xCpo.1              KLLGSLKSDV LCFLTIHSYG QLLLLPYGYT KDPSINHEEM INVAQKAAAK LQEKHGTEYR
xCpo.2              GLIEKTKSDI LCYLTIHSYG QMILLPYGYK KDPSPNHDEM MLVAKNAVAK MKEKHNNEYE
xCpo.3              EFVESRKADI VCFLTMHSYS QLILTAYGYS TGLSRNYNEI FKVAEMAASA MEKIHGTKYR
xCpo.4              NLMERTKSQI LFYLTIHSYG QYILLPYGST TNPSVNHVEM TKVAEAAAAK MKEKHNIVYT
bCPA1               DFVKD-HGNF KAFLSIHSYS QLLLYPYGYT TQSIPDKTEL NQVAKSAVEA LKSLYGTSYK
                                 H196

                           370        380        390        400        410        420
hCPO                VGSSADILYA SSGSSRDWAR DIGIPFSYTF ELRDSGTYGF VLPEAQIQPT CEETMEAVLS
xCpo.1              VGSTSHLLYS NSGSSRDWAT DLGINFSYTF ELRDTGAHGF ILPANQIRPT CEETMAGVMT
xCpo.2              YESSAVILYY DSGSSGDWTV ELGIQLSYTL ELRDNGTYGF VLPPDQIKPT CEETTTAVMS
xCpo.3              AGPFSKLLYE ASGTSQDWVH DLGIDFSFTF ELRDNGSHKF TLPEDQIQPT CEETMAGVMT
xCpo.4              VGSSSVVLYE NSGSSCDWAG DIGIKFSYTF ELRDNGTYGF QLPAELIKPT CEETMTAVIS
bCPA1               YGSIITTIYQ ASGGSIDWSY NQGIKYSFTF ELRDTGRYGF LLPASQIIPT AQETWLGVLT
                                X255             E270

                           430        440        450        460
hCPO                VLDDVYAKHW HSDSAGRVTS ATMLLGLLVS CMSLL----- -
xCpo.1              IVEHVDAKFF NSAI--S-IF SSN--LVSLS LIIGLYYAIF -
xCpo.2              MVEYINEEYL ENSG--V-TT TSFWLNVFLS FTVCIYYGIA K
xCpo.3              IIEYVNEKYF PNKAS--TTV FNCWINILIF NTFMQVSVLF F
xCpo.4              MMEYANEKYL ENSA--K-TV TFMWLNVLLS CAVCMYYALL H
bCPA1               IMEHTLNNLY ---------- ---------- ---------- -
```

Supplementary Figure S7: Raw data used in the preparation of Figures 6 and 7.

a. Raw data used in the preparation of Fig. 6a. Lanes of top western blot and bottom Ponceau S stain are aligned by sample.

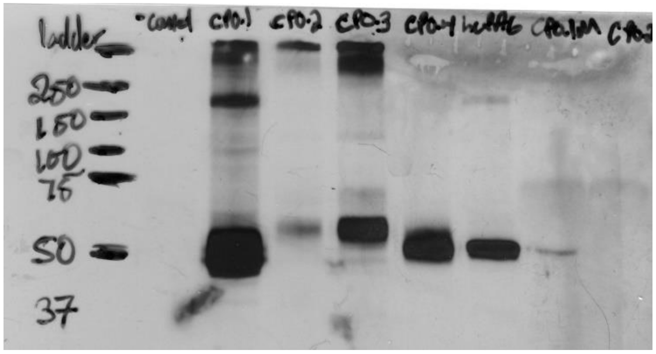

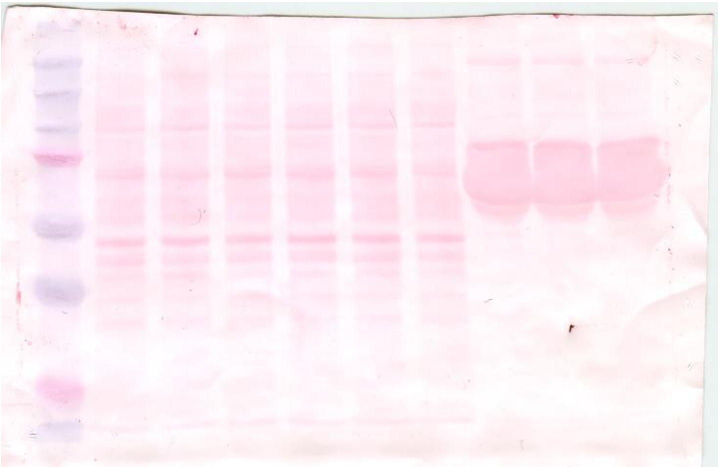

b. Raw data used in the preparation of Fig. 6c

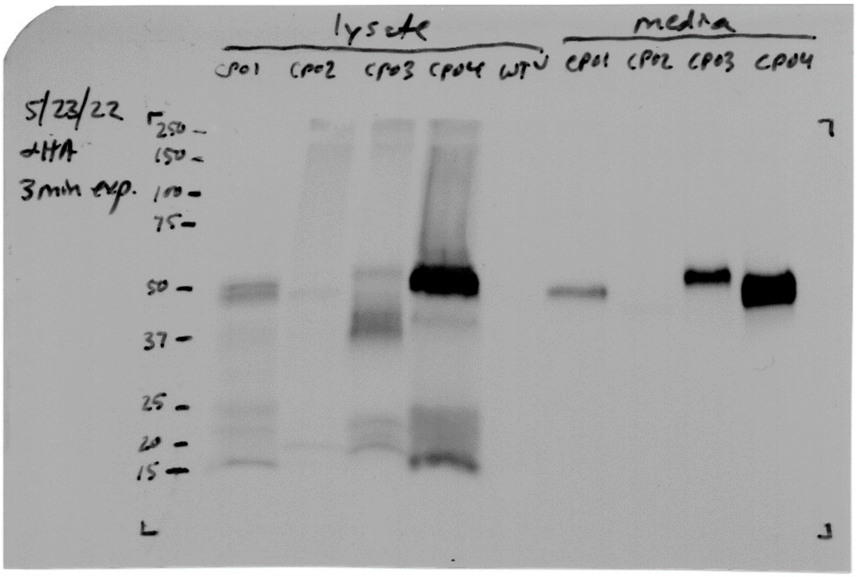

c. Raw data used in the preparation of Fig. 6d. Lanes of top western blot and bottom Ponceau S stain are aligned by sample.

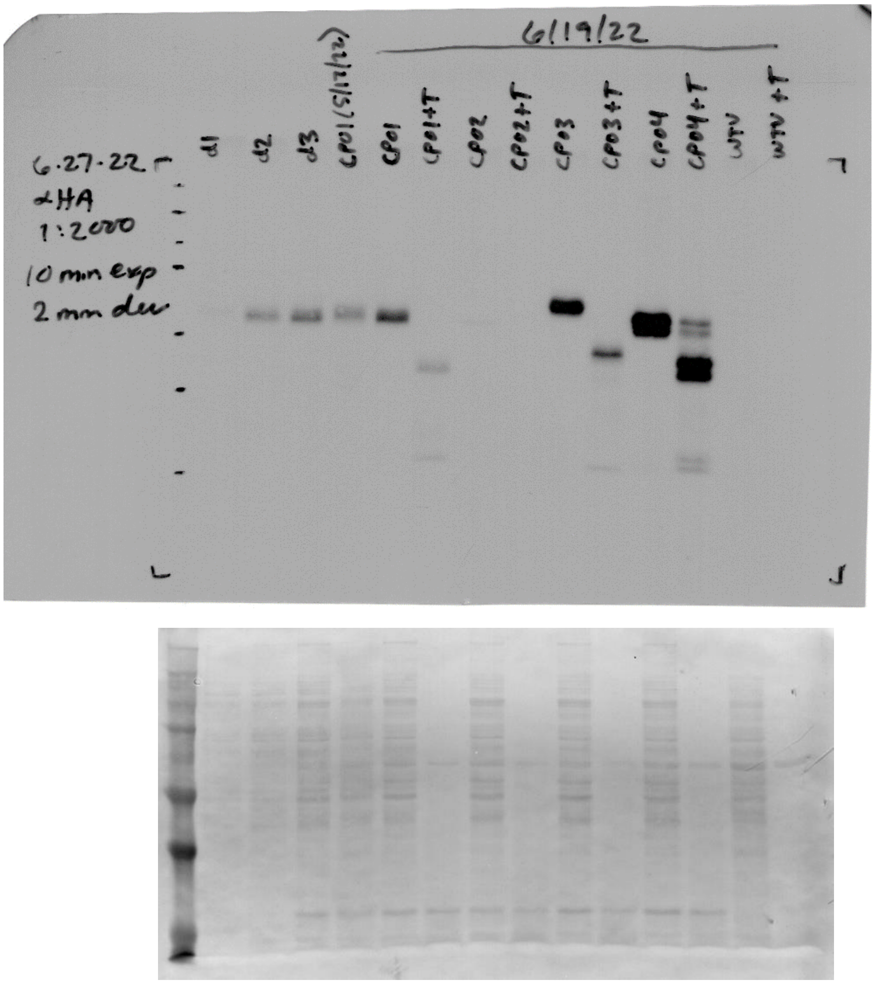

d. Raw data used in the preparation of Fig. 7a

Rate of reaction (slope		Trypsinized Media						
		WTV	CPO1	CPO2	CPO3	CPO4		
	FA-EE	0.049	-0.186	-0.004	-0.099	-0.161		
	FA-EE	0.120	-0.245	-0.034	-0.096	-0.317		
	FA-EE	0.068	-0.184	0.041	-0.086	-0.257		
	FA-EE	-0.080	-0.185	-0.313	-0.299			
	FA-EE		-0.164		-0.195			
	FA-EE		-0.125		-0.152			
average		0.039	-0.182	-0.078	-0.155	-0.245		
stdev		0.085	0.039	0.160	0.082	0.079		
	FA-FA	0.131	-0.195	-0.214	-0.293	-1.346		
	FA-FA	0.033	-0.076	-0.252	-0.218	-1.397		
	FA-FA	0.118	0.043	-0.285	-0.165	-1.355		
	FA-FA	-0.072	-0.110					
	FA-FA	-0.083						
	FA-FA	-0.257						
average		-0.022	-0.085	-0.250	-0.225	-1.366		
stdev		0.147	0.099	0.036	0.064	0.027		
	FA-FF	-0.162	-0.149	-0.294	-0.229	-0.462		
	FA-FF	-0.244	-0.090	-0.433	-0.082	-0.426		
	FA-FF	-0.134	-0.183	-0.098	-0.270	-0.481		
	FA-FF		-0.110			-0.646		
	FA-FF					-0.707		
average		-0.180	-0.133	-0.275	-0.194	-0.544		
stdev		0.057	0.041	0.168	0.099	0.124		
Raw Data								
average					stdev			
	FA-EE	FA-FA	FA-FF			FA-EE	FA-FA	FA-FF
WTV	0.039	-0.022	-0.180		WTV	0.085	0.147	0.057
CPO1	-0.182	-0.085	-0.133		CPO1	0.039	0.099	0.041
CPO2	-0.078	-0.250	-0.275		CPO2	0.160	0.036	0.168
CPO3	-0.155	-0.225	-0.194		CPO3	0.082	0.064	0.099
CPO4	-0.245	-1.366	-0.544		CPO4	0.079	0.027	0.124
Data Inverted and WTV subtracted								
average					stdev			
	FA-EE	FA-FA	FA-FF			FA-EE	FA-FA	FA-FF
WTV	0.000	0.000	0.000		WTV	0.085	0.147	0.057
CPO1	0.221	0.063	-0.047		CPO1	0.039	0.099	0.041
CPO2	0.117	0.229	0.095		CPO2	0.160	0.036	0.168
CPO3	0.194	0.204	0.014		CPO3	0.082	0.064	0.099
CPO4	0.284	1.344	0.364		CPO4	0.079	0.027	0.124

Supplementary Methods: *Xenopus tropicalis* CPO sequences

cpo.1 cDNA, HA tag inserted

```
>XM_031892508.1 PREDICTED: Xenopus tropicalis carboxypeptidase O gene 1
(cpo.1), transcript variant X1, mRNA
GAGTTCAGTAATTCATTAGAAATTTGATGCAAGCTAGCATTCAGGTTCTTCAAAAAACATCTTTTAATTC
ATTTTTTAAGGATGAAGCGCATACTTCTCTGCATTTGTATTTTAGGCATTCTAACACAGGTTGGGGCATA
CTTAAAGGTCCAGTACAATGGAGATCAGGTTTTGAAGCTCACCCTGAAAACAACAGATGACTGTGAGCAC
ATGCAAAATATCTGCAAACAACTCCAGCTTGATCTATGGAAGCCAAGCAGAATAGAAGATATTCAACCTG
GGAAAGAAATGCATGTTCGAATACCTTTTCCACTTCTTCAAAAATTCAAAGAGAACCTTCATCAATATTC
TATTCCATTCGAAGTCATGATAAAAGATGTACAAAAACTTATAGACAGCAGCAATGTGGGTGACTACAGA
AGACAGAAAAAAATATTGGCTGAATTTGATTATACCACATATCACCCCATGGATGAGATTTATCAGTGGA
TGGATCAAGTTAAAGAGGCATATAGTGACCTGGTTTCTATGCATTACCTAGGATCAACATATGAGCTAAG
GCCTATTTACTATTTCAAAATTGGCTGGCCTTCTGACAAACAGAAGAAGATCATTTGGATGGACTGTGGG
ATACATGCTCGTGAATGGATTGCCGTTGCCTATTGCCAATGGTTTGTTAAAGAGATTCTTGAAACTCACA
AGACAAATCCTTTGCTCCAGAAAGTACTCCATAACATAGACTTCTATATTGTACCTGTCCTAAATATTGA
TGGATTTGTTTATTCCTGGAATGTGAATCGTCTTTGGCGAAAATCGCGGTCTCCTCATAACAATGGATCC
TGCTACGGGGTGGACCTTAATCGAAATTTTAATTCAAAATGGGGCAGTATTGGCGCATCTAACAATTGTA
GAGATGAAACATACTGCGGAACTGGACCTGCATCTGAACCAGAAGTTAATGCTGTATCTAAGCTGCTGGG
GAGTCTTAAATCTGATGTTTTGTGTTTCCTAACAATTCATTCCTATGGTCAACTTCTTCTTCTGCCCTAT
GGTTATACAAAGGATCCTTCTATAAATCATGAAGAGATGATAAATGTTGCTCAGAAGGCTGCTGCAAAAC
TCCAAGAGAAGCATGGCACCGAGTACAGAGTTGGATCTACATCACACTTACTATATAGCAATTCTGGGTC
ATCTCGAGACTGGGCCACAGATCTAGGAATTAATTTTTCCTATACATTTGAGCTGAGAGACACTGGTGCA
CATGGATTTTATACTTCCTGCGAATCAGATCAGACCTACTTGTGAAGAAACCATGGCTGGAGTAATGACCA
TTGTTGAACATGTTGATGCA TAC CCA TAC GAT GTT CCA GAT TAC GCT
AAGTTCTTTAACAGTGCTATAAGCATTTTCTCTTCCAATCTTGTGAGTCT
GTCTCTCATCATTGGTCTTTATTATGCAATATTTTAATACCAGAGTGGTTTAGATGGTATAAATGTAGTT
ATGCATTCGTTCGACTGGATAAATGAGAAGCATCCATCTATTTACCCGTGTTGCCTTTTATTGTGTTGTT
TTCTTTTATTATTAAACATTAAACCGAGTGTAACTTATTAATGATAGCAAACATTACAGTTCTGTCACAA
TTTTCTTCACATTACCAAGTAAAGCATATGTTTGTCATGCA
```

Cpo.1 protein, HA tag inserted

```
>XP_031748368.1 carboxypeptidase O gene 1 isoform X1 [Xenopus tropicalis]
MKRILLCICILGILTQVGAYLKVQYNGDQVLKLTLKTTDDCEHMQNICKQLQLDLWKPSRIEDIQPGKEM
HVRIPFPLLQKFKENLHQYSIPFEVMIKDVQKLIDSSNVGDYRRQKKILAEFDYTTYHPMDEIYQWMDQV
KEAYSDLVSMHYLGSTYELRPIYYFKIGWPSDKQKKIIWMDCGIHAREWIAVAYCQWFVKEILETHKTNP
LLQKVLHNIDFYIVPVLNIDGFVYSWNVNRLWRKSRSPHNNGSCYGVDLNRNFNSKWGSIGASNNCRDET
YCGTGPASEPEVNAVSKLLGSLKSDVLCFLTIHSYGQLLLLPYGYTKDPSINHEEMINVAQKAAAKLQEK
HGTEYRVGSTSHLLYSNSGSSRDWATDLGINFSYTFELRDTGAHGFILPANQIRPTCEETMAGVMTIVEH
VDAYPYDVPDYAKFFNSAISIFSSNLVSLSLIIGLYYAIF
```

cpo.1 cDNA for cloning

```
>cpo.1_cloning
GAATTCTAAGGATGAAGCGCATACTTCTCTGCATTTGTATTTTAGGCATTCTAACACAGGTTGGGGCATACTTAAAG
GTCCAGTACAATGGAGATCAGGTTTTGAAGCTCACCCTGAAAACAACAGATGACTGTGAGCACATGCAAAATATCTG
CAAACAACTCCAGCTTGATCTATGGAAGCCAAGCAGAATAGAAGATATTCAACCTGGGAAAGAAATGCATGTTCGAA
TACCTTTTCCACTTCTTCAAAAATTCAAAGAGAACCTTCATCAATATTCTATTCCATTCGAAGTCATGATAAAAGAT
GTACAAAAACTTATAGACAGCAGCAATGTGGGTGACTACAGAAGACAGAAAAAAATATTGGCTGAATTTGATTATAC
CACATATCACCCCATGGATGAGATTTATCAGTGGATGGATCAAGTTAAAGAGGCATATAGTGACCTGGTTTCTATGC
ATTACCTAGGATCAACATATGAGCTAAGGCCTATTTACTATTTCAAAATTGGCTGGCCTTCTGACAAACAGAAGAAG
ATCATTTGGATGGACTGTGGGATACATGCTCGTGAATGGATTGCCGTTGCCTATTGCCAATGGTTTGTTAAAGAGAT
TCTTGAAACTCACAAGACAAATCCTTTGCTCCAGAAAGTACTCCATAACATAGACTTCTATATTGTACCTGTCCTAA
ATATTGATGGATTTGTTTATTCCTGGAATGTGAATCGTCTTTGGCGAAAATCGCGGTCTCCTCATAACAATGGATCC
```

TGCTACGGGGTGGACCTTAATCGAAATTTTAATTCAAAATGGGGCAGTATTGGCGCATCTAACAATTGTAGAGATGA
AACATACTGCGGAACTGGACCTGCATCTGAACCAGAAGTTAATGCTGTATCTAAGCTGCTGGGGAGTCTTAAATCTG
ATGTTTTGTGTTTCCTAACAATTCATTCCTATGGTCAACTTCTTCTTCTGCCCTATGGTTATACAAAGGATCCTTCT
ATAAATCATGAAGAGATGATAAATGTTGCTCAGAAGGCTGCTGCAAAACTCCAAGAGAAGCATGGCACCGAGTACAG
AGTTGGATCTACATCACACTTACTATATAGCAATTCTGGGTCATCTCGAGACTGGGCCACAGATCTAGGAATTAATT
TTTCCTATACATTTGAGCTGAGAGACACTGGTGCACATGGATTTATACTTCCTGCGAATCAGATCAGACCTACTTGT
GAAGAAACCATGGCTGGAGTAATGACCATTGTTGAACATGTTGATGCA**TACCCATACGATGTTCCAGATTACGCT**AA
GTTCTTTAACAGTGCTATAAGCATTTTCTCTTCCAATCTTGTGAGTCTGTCTCTCATCATTGGTCTTTATTATGCAA
TATTTTAAGCTT

cpo.2 cDNA, HA tag inserted
>XM_002937111.4 PREDICTED: Xenopus tropicalis carboxypeptidase O gene 2 (cpo.2), mRNA
CTCATATACTCTGAGTTCAAACTGATGCTCTAAGCAGGACAAGAACATAAAATATTTCACATAAAAGATG
ATTTTTTTACTCGGGACAATCTTCCTTCTTGGAGTTCAAGTGTATGAGGGATCTTGTTTAACAGTTCAGT
ACAATGGAGATAATGTGTTTAAAATCACTCCAGAGACATCAGAACATGCACAATATCTGCAAACTTTGGC
AAATGAATGGCTGCTTGACCTCTGGAGGCCACAAACAGTTGAGCAGATCCATGAAGGAAGCGATATTCAT
GTACAAATCCCATTTGCTTACATGGAACAGATGAAGCAAAAACTTCTTCAGCATTCTATTCCTTATGAGG
TCCTTATAAATGATGTTCAGAAACTTATAGACAGCAATACCGTGAGTGCACCCAAAATCCAAAAAGCATC
CTTAGAAAATTATGACTATACTAAATATCACCCAATGGATGAGATTTATAACTGGATGGACCTGATGAAA
GAAAAACACAGTGAAATTGTATCACAGCATTATATTGGATGCACATATGAACTGCGACCAATGTACTATT
TAAAAATTGGTTGGCCATCAGACAAGCAAAAGAAAATTTTCTTCATAGACTGTGGATTTCATGCAAGAGA
ATGGATTTCTGTGGCATTTTGCCAGTGGTTTGTAAATGAGATTGTTTCACATTACAAAACAGATGCCATA
CTGGCCAATGTCCTCAAGCAAGTAGACTTTTATGTGTTACCAGTGATGAATATTGATGGATATGTTTACA
CCTGGACAACGAACCGTTTGTGGAGAAAGAATCGGTCTCCACATGAAAATGGGACTTGCTATGGAGTCGA
CCTAAACAGGAACTTTGACTCTCAATGGTGCTCTATTGGAGCTTCCAGGGACTGTAACAGCAATACATTT
TGTGGTCCGGAGGCAGCATCTGAACCTGAAACCAAAGCATTGTCTGGTCTTATTGAAAAGACGAAATCTG
ACATATTATGTTACCTCACCATTCATTCCTACGGGCAGATGATTCTACTTCCATACGGTTACAAGAAGGA
CCCTTCCCCAAATCATGATGAAATGATGTTAGTTGCAAAGAATGCAGTAGCTAAAATGAAAGAAAAACAT
AACAATGAGTATGAATATGAATCTTCAGCTGTTATATTGTATTATGATTCTGGATCTTCAGGGGACTGGA
CTGTTGAACTTGGAATACAATTATCATACACATTGGAACTTAGGGATAATGGCACCTATGGCTTTGTGCT
CCCTCCTGATCAGATCAAGCCTACATGTGAAGAAACTACTACTGCTGTTATGTCCATGGTTGAATATATA
AATGAAGAATACCTTGAAAATAGTGGAGTGACAACGACTTCTTTTTGGCTAAATGTTTTTCTATCATTTA
CAGTTTGCATCTACTATGGCATAGCTAAA**TACCCATACGATGTTCCAGATTACGCT**
TAACTCTATGACAATTAAAATGATCTTGTAAAAATATTATT
ATATACACCTGCATGAGTACCATCATTTATTATTTAAAACTAAATCTCATTTTCTATTACATAATAAAAT
GTGTATGAGAGTGCA

Cpo.2 protein, HA tag inserted
>XP_002937157.1 carboxypeptidase O [Xenopus tropicalis]
MIFLLGTIFLLGVQVYEGSCLTVQYNGDNVFKITPETSEHAQYLQTLANEWLLDLWRPQTVEQIHEGSDI
HVQIPFAYMEQMKQKLLQHSIPYEVLINDVQKLIDSNTVSAPKIQKASLENYDYTKYHPMDEIYNWMDLM
KEKHSEIVSQHYIGCTYELRPMYYLKIGWPSDKQKKIFFIDCGFHAREWISVAFCQWFVNEIVSHYKTDA
ILANVLKQVDFYVLPVMNIDGYVYTWTTNRLWRKNRSPHENGTCYGVDLNRNFDSQWCSIGASRDCNSNT
FCGPEAASEPETKALSGLIEKTKSDILCYLTIHSYGQMILLPYGYKKDPSPNHDEMMLVAKNAVAKMKEK
HNNEYEYESSAVILYYDSGSSGDWTVELGIQLSYTLELRDNGTYGFVLPPDQIKPTCEETTTAVMSMVEY
INEEYLENSGVTTTSFWLNVFLSFTVCIYYGIAK**YPYDVPDYA**

cpo.2 cDNA for cloning
>cpo.2_cloning
GAATTCTAAAAGATGATTTTTTTACTCGGGACAATCTTCCTTCTTGGAGTTCAAGTGTATGAGGGATCTTGTTTAAC
AGTTCAGTACAATGGAGATAATGTGTTTAAAATCACTCCAGAGACATCAGAACATGCACAATATCTGCAAACTTTGG

CAAATGAATGGCTGCTTGACCTCTGGAGGCCACAAACAGTTGAGCAGATCCATGAAGGAAGCGATATTCATGTACAA
ATCCCATTTGCTTACATGGAACAGATGAAGCAAAAACTTCTTCAGCATTCTATTCCTTATGAGGTCCTTATAAATGA
TGTTCAGAAACTTATAGACAGCAATACCGTGAGTGCACCCAAAATCCAAAAAGCATCCTTAGAAAATTATGACTATA
CTAAATATCACCCAATGGATGAGATTTATAACTGGATGGACCTGATGAAAGAAAAACACAGTGAAATTGTATCACAG
CATTATATTGGATGCACATATGAACTGCGACCAATGTACTATTTAAAAATTGGTTGGCCATCAGACAAGCAAAAGAA
AATTTTCTTCATAGACTGTGGATTTCATGCAAGAGAATGGATTTCTGTGGCATTTTGCCAGTGGTTTGTAAATGAGA
TTGTTTCACATTACAAAACAGATGCCATACTGGCCAATGTCCTCAAGCAAGTAGACTTTTATGTGTTACCAGTGATG
AATATTGATGGATATGTTTACACCTGGACAACGAACCGTTTGTGGAGAAAGAATCGGTCTCCACATGAAAATGGGAC
TTGCTATGGAGTCGACCTAAACAGGAACTTTGACTCTCAATGGTGCTCTATTGGAGCTTCCAGGGACTGTAACAGCA
ATACATTTTGTGGTCCGGAGGCAGCATCTGAACCTGAAACCAAAGCATTGTCTGGTCTTATTGAAAAGACGAAATCT
GACATATTATGTTACCTCACCATTCATTCCTACGGGCAGATGATTCTACTTCCATACGGTTACAAGAAGGACCCTTC
CCCAAATCATGATGAAATGATGTTAGTTGCAAAGAATGCAGTAGCTAAAATGAAAGAAAAACATAACAATGAGTATG
AATATGAATCTTCAGCTGTTATATTGTATTATGATTCTGGATCTTCAGGGGACTGGACTGTTGAACTTGGAATACAA
TTATCATACACATTGGAACTTAGGGATAATGGCACCTATGGCTTTGTGCTCCCTCCTGATCAGATCAAGCCTACATG
TGAAGAAACTACTACTGCTGTTATGTCCATGGTTGAATATATAAATGAAGAATACCTTGAAAATAGTGGAGTGACAA
CGACTTCTTTTTGGCTAAATGTTTTTCTATCATTTACAGTTTGCATCTACTATGGCATAGCTAAA**TACCCATACGAT**
GTTCCAGATTACGCTTAAGCTT

cpo.3 cDNA, HA tag inserted

>XM_018097644.2 PREDICTED: Xenopus tropicalis carboxypeptidase O
(LOC100490370), transcript variant X2, mRNA
GATGCTGGCATGTCTCTGTTTTACAGATGCCTTAGCACAGCATCTATACCAGCTACTGTTCACACAGAAC
TGGATAGGATCCATGAAACAGACTGGAAACTGATTTATGCTGCATGAGCTTGTGTGATTTGGGCATAATG
GGCATCATTCAGGCATACTCTAGGGAGCCTACTGATGTGCTGACATCAGCCTTCCGTGCATCGTAGCTAA
CTGGCATACTAAAATGGAGACCAGCACAGATGACTGTGGGCAGCTGGCTCTCGCCAGCTCTAGCAGAGCA
AAGAACAGATTGAAGAGAAAGAAGTTGAAAGCAAAGGACAACTTCAGCTGCCACAAAATGCATCATCTCT
GGATTGTCTGGACTTTTGGAACTCTGACTTTGCAGGTGTGTTGCACTGGAACAGAGTATGATGGGGGCAA
CATTTTAGAGATTACCCCAGAAAGTGAGAAACAAGTGCAGTGCTTACAGAATATCCTTCAGTCCTGGCTG
CTAGATCTTTTGAAGCCTTTGCAGCCTGAAGACATCAATGTCAAGACTACAGTGCATGTCAGGATCCCAT
CCACTGCTTTACAGCTCGTGAAAGAGGACCTGCTTCATTGTTCACAATCTTTGGAAATTCTGACTGGCAA
TGTGAAATACATAGAGGAAGACAAAATAGATACCAAAGAAACCAGGAAGACCATCAATGAATATAACTAC
ACCACATATCATCCAATGAATGAGATTTATGACTGGATAAATGGCATAGCAAAAAAGCACAGTCAGTTTG
TGACACAACATCTTTTGGGTTTAACTTATGAGTCAAGACCGATGCAGTACCTAAAGATCAGTCAGCCGTC
TGAAAATCATAAAAAGATTGTTTGGATAGATTGTGGAATCCATGCAAGGGAATGGATTGCGCCAGCATTC
TGCCAATGGTTTGTGAAAGAGATTGTACAGAATTATCAAAATGATCAAAGGATTAGAAAAATCCTCCAGA
ATTTAGACATATACGTCTTGCCAGTTCTCAATATTGATGGGTATATCTACTCGTGGACAAAAGAAAGGCT
CTGGAGAAAAAATCGTTCTCAATATGGGAATGGAACCTGCTATGGAGTGGACCTTAACCGCAACTTTAAT
GTATCATGGTGCACTCATAGATCATCCACAAATTGCAGTTCCAACTCCTTCTGTGGAAGTTCACCTGTTT
CAGAACCAGAGACCAGGGCAGTGGTAGAGTTTGTGGAAAGCAGAAAGGCAGATATTGTTTGCTTCCTAAC
AATGCACTCATACAGCCAACTCATCCTGACTGCTTATGGATACTCTACAGGGCTATCAAGGAATTATAAT
GAAATATTCAAAGTAGCTGAAATGGCAGCATCAGCCATGGAAAAAATACATGGTACAAAGTACAGGGCTG
GTCCATTTTCCAAACTCCTATATGAAGCTTCTGGAACATCCCAAGACTGGGTTCATGATCTAGGCATAGA
CTTCTCCTTCACCTTTGAACTGAGAGATAATGGTAGCCACAAGTTTACTTTGCCTGAGGACCAAATACAA
CCAACCTGTGAAGAGACTATGGCTGGTGTGATGACCATTATAGAATATGTCAATGAAAAATACTTT **TAC CCA**
TAC GAT GTT CCA GAT TAC GCT CCAA
ACAAGGCTTCGACCACTGTTTTTAATTGTTGGATAAATATCCTTATTTTCAACACATTTATGCAAGTTTC
AGTTTTATTTTTCTAACATTTCAAAAGGAAATATTAATCTTTTCCAGGTTGTCTACTCACATCCTTCCTC
CATGCTATTTTGCATAATACCCATAAATTAAATCAAATTACTCAAGAGGATGGTGTTCTGGGAGAAATAA
CTTTACTTTACAACAATAAGCTGGGTTTTCAATTGTTTCTATTTAAAGGGTCAACATATCTCCATTTTTA
ACATGAGTTCAATAAATATAGCTTGTGATAATATAAACATATTTCTTGCCTGTTGATTTAATCAAAAGAA
ATCTATGTTTTTGTTATTTCTTGACCTAAGCAAGGCAAAGAGTTTAGGCAAAAAGAAAATTAAACCTACT
CCATGTGTGATTTTTGCAAGATAGATAATTAAATTACCAGTGCACAGATAAACAGAAGTCAGTTTTATAG
GGGGTTGAATTATTCAGACTTTGTAAAAATAAAATGGAATCAATCAAGCCACCTTTTGATCAAGTCTACC

ACCTCTGGATTCCAGATCTCAATGAAGATGTGGGAGGAAGCCATAGACACTGCCTATAATTTATTAATCC
CAATAAAAGACAGATTTATACAATACAAGTTTCTCTAAATTGATATAACCCCTCTTAAGCTGAAAAGGTT
TGGCAAAAAACAGGATAACCTGTGTCCCTGATGTAAATCCCCAGGTGCTAACTTTATTCATATGATTTGT
ATTGTCCCCCTATAAAAGAATTGTGTGCTTCTGTAATGGAGGTCCTGGCCGCTGAACTGGGTACACCACA
AATTGCTGACCCCCTGCTCTGCTTACAAGGGGAAAAAGACCACATACTACCCACCAGTGTCACCAGAATT
CAGGTGCGATTCCCTCATGTTCTACATCAAGAAGGCGGTGATAATGCACTGGAAGGGAGATAACCCCCCT
CCATAACTTTCTGGAGGCAACTAGTAGACATGCACTGCTGTTTATTAAAGGCAAACTAAAGCTTAACTAA
AGAATTTGGCTAGAAATGTTATAAACTATGTTTTGGGCTCTTGTTCCAGCACAAGCCAACCACTGACTTT
TTTGCACTGAAGATCTGTGCCCCCAAGATGCCCCAATAGCTCCCCATCTCCTTTTCTTCTAATTCACTGC
ACATACTCTGTGCTACTGTCAGTTACTGAGCTTAGGGACAAATTCACAATATACTGTATATATAGAATAT
AAATGTCACAATATAAGACTGATTAGTAAATAATATGGATTATTACTGCATGACCAAAAGCAATTATCAG
AAATGAATAATCAACCCCGCAGCATTATTTTCTGCTTGATATTTGCAATGACCCCTGTGGCAACTCTGAG
GTACTGAATCATTACAACTGTAGAGATGCTGAACCTTTAGGCTGGGTCAATGAGTTCATTATATACAATA
TGACATTTCTAGCCATATTAATTTTTAGGGTTTAGTTCTCCTTTAAATTAATGTTCAAAATAAGGGTAGC
CAATTTCAGTTTGACAAGGTGTGGGTTGCTTGGTGTAATCCAGACTTTCTGGAGCTGTAATCCTTTCTTT
CAGGGTGTACGTGACCTGCATCTCACAGTTATAGAATCCTTCTGTGTACTTAAGCACCTGCTACAATGAG
CAATAATATTCTGCTGTCTGTTCTAGACTGTGGTTGAGCCAGAACCTATCAATTCTACTGTACATGATAG
TGACTGTTCATTGTTCTATAATTTGTCTTGTTTTGTCTGCTTTGTTTTGGGTAAAATCAATAAAACTTAC
CTTTAAATACAGGGGGATGGTCTGTGCCCTGGAATTCTAATTATATAGATCAGATGTGGTTTTGCAGAAC
AATTTAGTTATTTATGTATGCATCAGATATAAGTGAAAGGGTTGCAGAATTGTGCCCCCTAGCTAACCAA
TAAATACACCTTGTTTGTGTAGCTATATCCTGAGTGCACACAGCATTCTAATGGTCTAAATTCTATTTTG
ATATTAGTTGCTATACTTCCTCCAAATGAGTTTGCCGAAGATGTTATCATGCTCCTTATTCATTTTTCTT
TTATGTTTTGATTGTGTTTTGGGTTCTTTTTTAATTTGTGATGTCAATTGATCAGTTGTTTTGCTGTTTA
AAACATGTACTTCCTTCTCTGTTTATGCCTATGATTAAGTTCACATGTGCAGGAAACGCGTCAGGCGTAT
TCATTTTAAAGAGATGAATAAAGTTGAGATTTAAAGGACATCTTATGACATTCTGATGTGTGA

Cpo.3 protein, HA tag inserted
>XP_017953133.2 carboxypeptidase O isoform X2 [Xenopus tropicalis]
MHHLWIVWTFGTLTLQVCCTGTEYDGGNILEI
TPESEKQVQCLQNILQSWLLDLLKPLQPEDINVKTTVHVRIPSTALQLVKEDLLHCSQSLEILTGNVKYI
EEDKIDTKETRKTINEYNYTTYHPMNEIYDWINGIAKKHSQFVTQHLLGLTYESRPMQYLKISQPSENHK
KIVWIDCGIHAREWIAPAFCQWFVKEIVQNYQNDQRIRKILQNLDIYVLPVLNIDGYIYSWTKERLWRKN
RSQYGNGTCYGVDLNRNFNVSWCTHRSSTNCSSNSFCGSSPVSEPETRAVVEFVESRKADIVCFLTMHSY
SQLILTAYGYSTGLSRNYNEIFKVAEMAASAMEKIHGTKYRAGPFSKLLYEASGTSQDWVHDLGIDFSFT
FELRDNGSHKFTLPEDQIQPTCEETMAGVMTIIEYVNEKYF**YPYDVPDYA**PNKASTTVFNCWINILIFNTFMQVSVL
FF

cpo.3 cDNA for cloning

>cpo.3_cloning
CTCGAGCAAAATGCATCATCTCTGGATTGTCTGGACTTTTGGAACTCTGACTTTGCAGGTGTGTTGCACTGGAACAG
AGTATGATGGGGGCAACATTTTAGAGATTACCCCAGAAAGTGAGAAACAAGTGCAGTGCTTACAGAATATCCTTCAG
TCCTGGCTGCTAGATCTTTTGAAGCCTTTGCAGCCTGAAGACATCAATGTCAAGACTACAGTGCATGTCAGGATCCC
ATCCACTGCTTTACAGCTCGTGAAAGAGGACCTGCTTCATTGTTCACAATCTTTGGAAATTCTGACTGGCAATGTGA
AATACATAGAGGAAGACAAAATAGATACCAAAGAAACCAGGAAGACCATCAATGAATATAACTACACCACATATCAT
CCAATGAATGAGATTTATGACTGGATAAATGGCATAGCAAAAAAGCACAGTCAGTTTGTGACACAACATCTTTTGGG
TTTAACTTATGAGTCAAGACCGATGCAGTACCTAAAGATCAGTCAGCCGTCTGAAAATCATAAAAAGATTGTTTGGA
TAGATTGTGGAATCCATGCAAGGGAATGGATTGCGCCAGCATTCTGCCAATGGTTTGTGAAAGAGATTGTACAGAAT
TATCAAAATGATCAAAGGATTAGAAAAATCCTCCAGAATTTAGACATATACGTCTTGCCAGTTCTCAATATTGATGG
GTATATCTACTCGTGGACAAAAGAAAGGCTCTGGAGAAAAAATCGTTCTCAATATGGGAATGGAACCTGCTATGGAG
TGGACCTTAACCGCAACTTTAATGTATCATGGTGCACTCATAGATCATCCACAAATTGCAGTTCCAACTCCTTCTGT
GGAAGTTCACCTGTTTCAGAACCAGAGACCAGGGCAGTGGTAGAGTTTGTGGAAAGCAGAAAGGCAGATATTGTTTG
CTTCCTAACAATGCACTCATACAGCCAACTCATCCTGACTGCTTATGGATACTCTACAGGGCTATCAAGGAATTATA
ATGAAATATTCAAAGTAGCTGAAATGGCAGCATCAGCCATGGAAAAAATACATGGTACAAAGTACAGGGCTGGTCCA
TTTTCCAAACTCCTATATGAAGCTTCTGGAACATCCCAAGACTGGGTTCATGATCTAGGCATAGACTTCTCCTTCAC

CTTTGAACTGAGAGATAATGGTAGCCACAAGTTTACTTTGCCTGAGGACCAAATACAACCAACCTGTGAAGAGACTA
TGGCTGGTGTGATGACCATTATAGAATATGTCAATGAAAAATACTTT**TACCCATACGATGTTCCAGATTACGCT**CCA
AACAAGGCTTCGACCACTGTTTTTAATTGTTGGATAAATATCCTTATTTTCAACACATTTATGCAAGTTTCAGTTTT
ATTTTTCTAAGAATTC

cpo.4 cDNA, HA tag inserted
>XM_018097354.2 PREDICTED: Xenopus tropicalis carboxypeptidase O
(LOC100489361), mRNA
CCTGCGCAAACTTACAGTTGGTAAGAAACCATGAAGCTTTTCAACTTGTGGTTCTGTCTGCTGGGAATCC
TAGTATATGAAGGATTCTGTATGAAGGTGAAATATGATGGTGATCAGGTTTTAAAAATGATCCCTCAGAC
ACTGAAACATGCCCAGTTTATGCAAGGCTTAATTCAGGAATGGATGCTTGATTTATGGAAACCAGTTATG
GTGGAACAAATCCAAGCAGGAAGAGAGATGCATGTCCGGGTACCATTTTCCCATCTGCAAGAAATAAAAG
AGAAGCTTTTGCAAAACATGCTACCATATCAAATCCTAATCAGTGATGTGCAGGAACTGGTCAACAGGAA
TACACCAATTGAGACAAAAATGCAAAAAATATCATTGGACAATTACGATTATACTAAATATCATCCAATG
GATGAGATATATGATTGGATGGAACAAATTCAGCTGAAACACAGGGACCTGGTAACAAAACATTTTATGG
GATCTACATATGAACTGCGGCCAATCTATTACTTTAAAATTGGATGGCCGTCAGACAAACCAAAGAAAAT
AATCTTCATGGACTGTGGAATCCATGCTAGAGAGTGGATTGCAGTTGCTTACTGTCAATGGTTTGTGAAA
GAGATCCTTTCAAGCCACAGTAACAACAAATTACTAACCAATGTTCTCAAACAAGTAGACTTCTATGTAG
TACCAGTGTTTAATATTGATGGATATATTTACTCCTGGACAACAGAGCGCCTGTGGAGAAAAAATCGCTC
ACCACATAATAATGCCACATGCTATGGAGTGGACCTGAATCGTAACTTTAACTCATCATGGTGCAGTGTT
GGTGCTTCCAGAGATTGCAATTCCCAAACTTTCTGTGGCTCAGCCCCTGCTTCAGAACCTGAGACTCAGG
CAGTTGCCAATCTTATGGAAAGAACAAAATCTCAAATTCTTTTCTATCTTACCATTCACTCCTATGGACA
ATATATTCTCCTTCCTTATGGTTCCACAACAAATCCTTCAGTAAACCATGTGGAAATGACAAAAGTTGCA
GAAGCCGCTGCTGCTAAAATGAAGGAAAAACATAACATCGTGTACACAGTGGGCTCATCATCTGTCGTAT
TGTATGAAAACTCAGGATCCTCATGTGACTGGGCTGGTGATATTGGAATAAAATTCTCTTACACCTTTGA
ACTGAGAGACAATGGCACATATGGTTTTCAGCTTCCAGCTGAACTGATAAAGCCTACTTGTGAAGAGACA
ATGACTGCAGTAATATCTATGATGGAGTATGCCAACGAAAAA**TAC CCA TAC GAT GTT CCA GAT TAC**
GCTTACCTTGAGAATAGTGCAAAAACTGTGA
CTTTTATGTGGCTAAATGTGCTTCTTTCTTGTGCTGTTTGCATGTATTATGCATTACTGCATTAACCACA
CCAATTCTGAAAAATTTGTTTTAACAAGATTGTCTTCTTTTTAAAGAAAATTAAGTGTCCTTGTAATCTG
AAATAATTCACACATTTAGAAGGGTTTTTGGGGGTCTAATAAATGCCTGTTTGTTACAAA

Cpo.4 protein, HA tag inserted
>XP_017952843.2 carboxypeptidase O [Xenopus tropicalis]
MKLFNLWFCLLGILVYEGFCMKVKYDGDQVLKMIPQTLKHAQFMQGLIQEWMLDLWKPVMVEQIQAGREM
HVRVPFSHLQEIKEKLLQNMLPYQILISDVQELVNRNTPIETKMQKISLDNYDYTKYHPMDEIYDWMEQI
QLKHRDLVTKHFMGSTYELRPIYYFKIGWPSDKPKKIIFMDCGIHAREWIAVAYCQWFVKEILSSHSNNK
LLTNVLKQVDFYVVPVFNIDGYIYSWTTERLWRKNRSPHNNATCYGVDLNRNFNSSWCSVGASRDCNSQT
FCGSAPASEPETQAVANLMERTKSQILFYLTIHSYGQYILLPYGSTTNPSVNHVEMTKVAEAAAAKMKEK
HNIVYTVGSSSVVLYENSGSSCDWAGDIGIKFSYTFELRDNGTYGFQLPAELIKPTCEETMTAVISMMEY
ANEK**YPYDVPDYA**YLENSAKTVTFMWLNVLLSCAVCMYYALLH

cpo.4 cDNA for cloning
>cpo.4_cloning
CTCGAGAAACCATGAAGCTTTTCAACTTGTGGTTCTGTCTGCTGGGAATCCTAGTATATGAAGGATTCTGTATGAAG
GTGAAATATGATGGTGATCAGGTTTTAAAAATGATCCCTCAGACACTGAAACATGCCCAGTTTATGCAAGGCTTAAT
TCAGGAATGGATGCTTGATTTATGGAAACCAGTTATGGTGGAACAAATCCAAGCAGGAAGAGAGATGCATGTCCGGG
TACCATTTTCCCATCTGCAAGAAATAAAAGAGAAGCTTTTGCAAAACATGCTACCATATCAAATCCTAATCAGTGAT
GTGCAGGAACTGGTCAACAGGAATACACCAATTGAGACAAAAATGCAAAAAATATCATTGGACAATTACGATTATAC
TAAATATCATCCAATGGATGAGATATATGATTGGATGGAACAAATTCAGCTGAAACACAGGGACCTGGTAACAAAAC
ATTTTATGGGATCTACATATGAACTGCGGCCAATCTATTACTTTAAAATTGGATGGCCGTCAGACAAACCAAAGAAA

ATAATCTTCATGGACTGTGGAATCCATGCTAGAGAGTGGATTGCAGTTGCTTACTGTCAATGGTTTGTGAAAGAGAT
CCTTTCAAGCCACAGTAACAACAAATTACTAACCAATGTTCTCAAACAAGTAGACTTCTATGTAGTACCAGTGTTTA
ATATTGATGGATATATTTACTCCTGGACAACAGAGCGCCTGTGGAGAAAAAATCGCTCACCACATAATAATGCCACA
TGCTATGGAGTGGACCTGAATCGTAACTTTAACTCATCATGGTGCAGTGTTGGTGCTTCCAGAGATTGCAATTCCCA
AACTTTCTGTGGCTCAGCCCCTGCTTCAGAACCTGAGACTCAGGCAGTTGCCAATCTTATGGAAAGAACAAAATCTC
AAATTCTTTTCTATCTTACCATTCACTCCTATGGACAATATATTCTCCTTCCTTATGGTTCCACAACAAATCCTTCA
GTAAACCATGTGGAAATGACAAAAGTTGCAGAAGCCGCTGCTGCTAAAATGAAGGAAAAACATAACATCGTGTACAC
AGTGGGCTCATCATCTGTCGTATTGTATGAAAACTCAGGATCCTCATGTGACTGGGCTGGTGATATTGGAATAAAAT
TCTCTTACACCTTTGAACTGAGAGACAATGGCACATATGGTTTTCAGCTTCCAGCTGAACTGATAAAGCCTACTTGT
GAAGAGACAATGACTGCAGTAATATCTATGATGGAGTATGCCAACGAAAAA**TACCCATACGATGTTCCAGATTACGC**
TTACCTTGAGAATAGTGCAAAAACTGTGACTTTTATGTGGCTAAATGTGCTTCTTTCTTGTGCTGTTTGCATGTATT
ATGCATTACTGCATTAAGAATTC
